# The HOPS and vCLAMP protein Vam6 connects polyphosphate with mitochondrial function and oxidative stress resistance in *Cryptococcus neoformans*

**DOI:** 10.1128/mbio.00328-25

**Published:** 2025-02-25

**Authors:** Eddy Sánchez-León, Kabir Bhalla, Guanggan Hu, Christopher W. J. Lee, Melissa Lagace, Won Hee Jung, James W. Kronstad

**Affiliations:** 1 The Michael Smith Laboratories, Department of Microbiology and Immunology, University of British Columbia, Vancouver, Canada; 2 Department of Microbiology and Immunology, University of British Columbia, Vancouver, Canada; 3 Department of Systems Biotechnology, Chung-Ang University, Anseong, South Korea; Institut Pasteur, Paris, France

**Keywords:** Vam6, Vtc2, mitochondria, vacuole, oxidative stress, polyphosphate

## Abstract

*Cryptococcus neoformans* is considered one of the most dangerous fungal threats to human health, and the World Health Organization recently ranked it in the critical priority group for perceived public health importance. Proliferation of *C. neoformans* within mammalian hosts is supported by its ability to overcome nutritional limitations and endure stress conditions induced by the host immune response. Previously, we reported that the Vam6/Vps39/TRAP1-domain protein Vam6 was crucial for vacuolar morphology, iron acquisition, and virulence. However, the molecular mechanisms underlying the pleiotropic phenotypes resulting from loss of Vam6 remain poorly understood. In this study, we determined that Vam6 has roles in the HOPS complex for endomembrane trafficking to the vacuole and in the vCLAMP membrane contact site between the vacuole and mitochondria. Importantly, both of these roles regulate polyphosphate (polyP) metabolism, as demonstrated by a defect in trafficking of the VTC complex subunit Vtc2 for polyphosphate synthesis and by an influence on mitochondrial functions. In the latter case, Vam6 was required for polyP accumulation in response to electron transport chain inhibition and for overcoming oxidative stress. Overall, this work establishes connections between endomembrane trafficking, mitochondrial functions, and polyP homeostasis in *C. neoformans*.

**IMPORTANCE:**

A detailed understanding of stress resistance by fungal pathogens of humans may provide new opportunities to improve antifungal therapy and combat life-threatening diseases. Here, we used a *vam6* deletion mutant to investigate the role of the homotypic fusion and vacuole protein sorting (HOPS) complex in mitochondrial functions and polyphosphate homeostasis in *Cryptococcus neoformans*, an important fungal pathogen of immunocompromised people including those suffering from HIV/AIDS. Specifically, we made use of mutants defective in late endocytic trafficking steps to establish connections to oxidative stress and membrane trafficking with mitochondria. In particular, we found that mutants lacking the Vam6 protein had altered mitochondrial function, and that the mutants were perturbed for additional mitochondria and vacuole-related phenotypes (e.g., membrane composition, polyphosphate accumulation, and drug sensitivity). Overall, our study establishes connections between endomembrane trafficking components, mitochondrial functions, and polyphosphate homeostasis in an important fungal pathogen of humans.

## INTRODUCTION

Diseases caused by fungal pathogens result in the deaths of >1.5 million people each year, and yet the development of novel antifungal agents and effective therapies has not advanced sufficiently to mitigate the negative impact on vulnerable people (1, 2). *Cryptococcus* species are considered along with *Candida* spp. and *Aspergillus fumigatus* as some of the most dangerous fungi for humans, causing severe systemic mycoses which frequently result in mortality (1). For example, *Cryptococcus neoformans* is responsible for nearly 180,000 cases of meningoencephalitis per year and ~19% of HIV/AIDS-related deaths (3, 4). *C. neoformans* is also a useful model for investigating mechanisms of fungal pathogenesis because of its genetic tractability. In this regard, we are employing the pathogen to investigate the roles of endocytic trafficking in virulence, including recent characterization of Vam6/Vps39/TRAP1-domain proteins (5). Mutants lacking these proteins (Vam6 and Vps3) were unable to cause disease in a mouse model of cryptococcosis and had a reduced ability to use heme as a source of iron (5). Vps3 and Vam6 are integral subunits of the CORVET (class C core endosomal vacuole tethering) and HOPS (homotypic fusion and protein sorting) complexes, respectively, and are known in other model systems to play crucial roles in early and late endocytic trafficking and vacuolar fusion (6–10). Our recent analysis highlights how disruptions in membrane trafficking can impair the ability of *C. neoformans* to acquire and transport essential nutrients, thereby reducing virulence.

In general, the endocytic machinery also supports organelle functions through vesicle trafficking and membrane contact sites (MCS), functions crucial for the transport of ions, metabolites, protein cargo, and lipids to ensure proper cellular homeostasis (8–10). In *Saccharomyces cerevisiae*, for example, the formation of an MCS between the vacuole and mitochondria (vCLAMPs) is assisted by the independent interaction of the HOPS complex subunit Vam6/Vps39 with the Rab GTPase Ypt7 and the translocase of the outer membrane (TOM) subunit Tom40 (11). This study found that vCLAMPs are important for cell survival under starvation conditions or stress. Vam6 also has roles independent of the HOPS complex or vCLAMP formation, such as participating in the ethanolamine-stimulated phosphatidylethanolamine (PE) trafficking to the mitochondria (12). The importance of regulating the metabolism of PE is demonstrated by defective mitochondrial function and morphology in mutant cells impaired for the phosphatidylserine decarboxylase (Psd) pathway (13, 14).

Considering the diverse and essential functions of Vam6, detailed insights have emerged about its roles in endomembrane trafficking and mitochondrial and vacuolar function. In *Candida albicans*, a deletion mutant lacking Vam6 is impaired in the formation of large vacuoles, which affected hyphal development and penetration (7). Another study revealed that under oxidative stress conditions, the loss of Vam6 led to defective hyphal development and alterations in mitochondrial and vacuolar morphology, suggesting its critical role in maintaining organelle physiology under cellular stress (15). The importance of Vam6 is underscored by several studies linking mitochondrial functions with aspects of virulence for *C. neoformans* (16). For example, the modification of mitochondria morphology has been associated with a response to host conditions during an infection, where exposure to oxidative stress is a major challenge to fungal cells (17, 18). Dysfunctional late endomembrane trafficking processes not only interfere with mitochondria physiology but also impair vacuolar structure and function. From our previous study with Vam6 deletion mutants, we identified defects in vacuolar morphology, suggesting the role of this protein in vacuole biogenesis and/or stability (5). The vacuole phenotypes found in *vam6*Δ mutants resembled the *S. cerevisiae* type Class B/C vacuolar phenotypes found in *vps39* mutants (19, 20). Disruption of vacuolar morphological modifications impacts *C. neoformans* virulence and survival under environmental stresses (e.g., osmotic, temperature, starvation) highlighting the importance of this organelle in fungal pathogenicity (5, 21, 22).

In this study, we discovered that Vam6 influences the accumulation of polyphosphate (polyP) and its localization in cytoplasmic granules. Our analysis is consistent with observations in *S. cerevisiae* that polyP levels are influenced by trafficking and vacuolar functions (23). It is notable that polyP is associated with dense granules in platelets, and the formation of these granules requires endomembrane trafficking functions including the HOPS complex (24–26). Given that platelets have important functions in controlling bleeding, deficiencies in trafficking functions can lead to specific human disorders such as Hermansky-Pudlak syndrome (26). Our previous studies on polyP established its relevance in growth, cell surface architecture and size, mitochondrial function, and stress response in *C. neoformans,* findings consistent with activities in other organisms (27–34). Given this association and the fact that polyP is associated with mitochondria, we examined the impact of loss of Vam6 on mitochondrial phenotypes and found altered morphology and reduced growth on non-fermentable carbon sources. Disruptions in the homeostasis of major mitochondrial phospholipids and polyphosphate content were also associated with dysfunctional mitochondria, aberrant distribution of the vacuolar transporter chaperone (VTC) complex subunit Vtc2, and the damaging effect of growth with high levels of metal ions. Remarkably, these studies led to the discovery that inhibition of the electron transport chain (ETC) resulted in hyperaccumulation of polyP. This finding is consistent with reduced activity of the ETC being associated with lower membrane potential and higher levels of reactive oxygen species (ROS) (32). Consistent with a mitochondrial impact of Vam6, our analysis revealed an enhanced sensitivity to oxidative stress resulting in growth defects and hyperaccumulation of ROS in the *vam6*Δ mutant. Overall, we uncovered links between the endomembrane trafficking machinery, mitochondrial function, and polyP homeostasis in *C. neoformans*.

## RESULTS

### A mutant lacking Vam6 exhibits reduced polyphosphate

**Fig 1:**
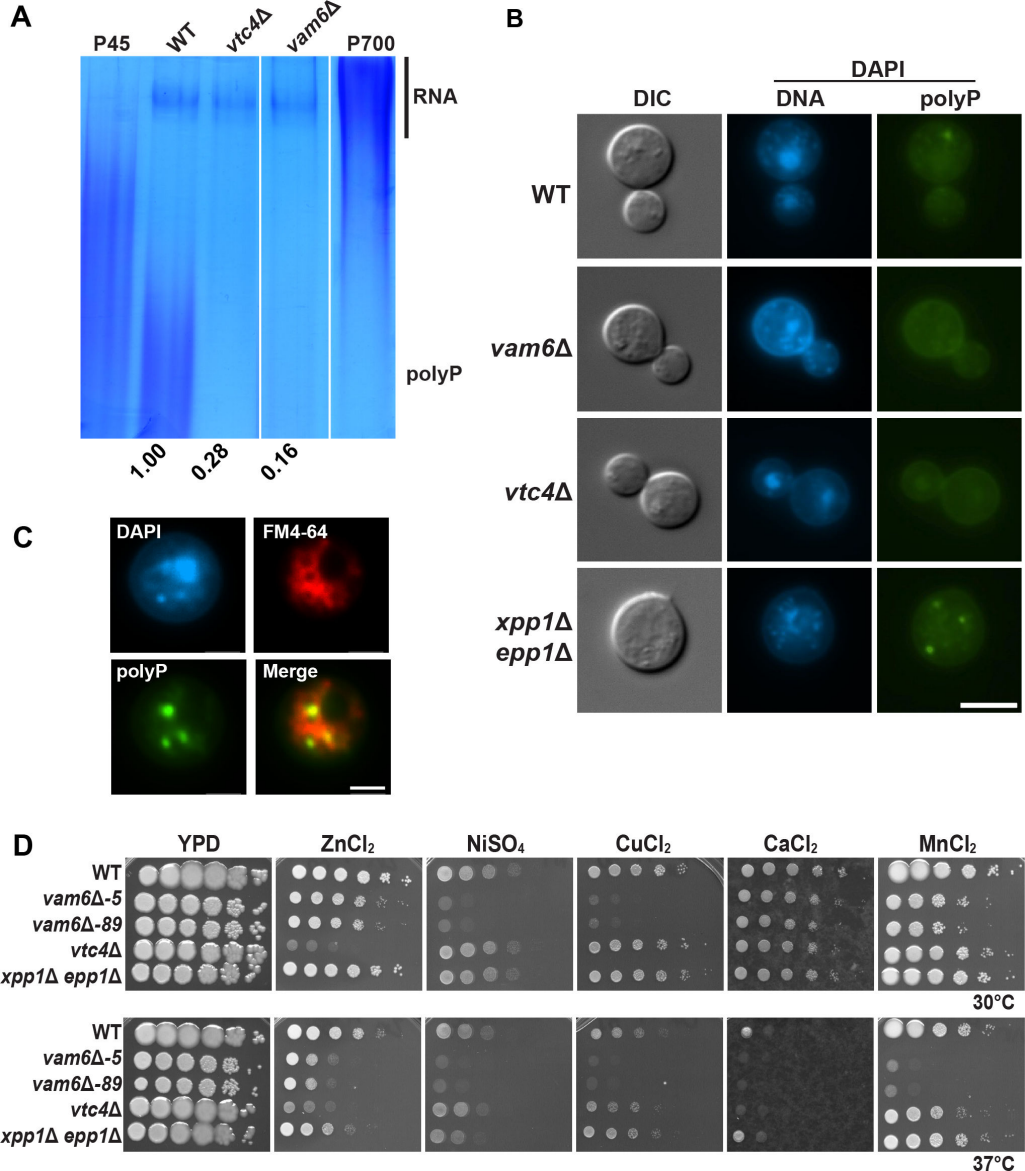
Trafficking mutants exhibit reduced inorganic polyP content and reduced tolerance to divalent metal ions. (**A**) Detection of polyP on a native acrylamide gel stained with toluidine blue O. Total RNA extracts (10 µg) from whole cell lysates of three biological replicates previously grown on YPD. Samples were loaded on the gels using polyP types 45 and 700 (P45 and P700, 10 µg) as standards. The numbers indicate densitometry measurements of the regions containing polyP normalized to the wild-type control region. The acrylamide gel is representative of at least three independent experiments and was spliced for labeling purposes. (**B**) Wide-field fluorescence microscopy showing representative images of the indicated strains of at least three independent experiments, each observing more than 100 cells. Cells were stained with DAPI (100 µg/mL) for 30 min at room temperature before imaging. Images were captured with DAPI (Ex/Em 359/461 nm) filter sets for DNA and the BrightLine (Ex/Em 407/530 nm) full multiband filter set for polyP. DIC indicates differential interference contrast microscopy. Scale bar, 5 µm. (**C**) Laser scanning confocal microscopy showing a wild-type strain stained with FM4-64 (5 µM) and DAPI (100 µg/mL) for 30 min and imaged as described above. FM4-64 images were captured with filter sets (Ex/Em 572/645 nm). Scale bar, 2 µm. (**D**) Spot assays of 10-fold serial dilutions of the indicated strains on YPD medium with or without zinc chloride (ZnCl_2_, 2.5 mM), nickel sulfate (NiSO_4_, 3 mM), copper chloride (CuCl_2_, 10 mM), calcium chloride (CaCl_2_, 0.25 M), or manganese chloride (MnCl_2_, 1.875 mM). The plates were incubated at 30°C or 37°C for 2–3 days before being photographed. Images are representative of at least three independent experiments.

We and others have found that loss of Vam6 impairs vacuolar functions and virulence in *C. neoformans* (5, 33). One aspect of the vacuole that is underexplored in pathogenic fungi involves the synthesis of polyP, as characterized in *S. cerevisiae*. Given the potential role of the HOPS complex in the formation of dense granules containing polyP in mammalian platelet cells, we hypothesized that a *vam6*Δ mutant would have a defect in polyP synthesis and localization. We therefore visualized RNA and polyP from whole cell lysates on polyacrylamide gels stained with toluidine blue O (Fig. 1A; Fig. S1A). We found that, compared to the wild-type strain, the *vam6*Δ mutant had reduced polyP levels that were similar to a *vtc4*Δ mutant defective in polyphosphate polymerase (34). We also stained the cells with a high concentration of 4´,6-diamidino-2-phenylindole (DAPI) and examined the presence of polyP granules by fluorescence microscopy. In contrast to the blue fluorescence (maximum emission of 456 nm) that results from DAPI staining of DNA, polyP staining gives an emission fluorescence shift with a maximum wavelength of 526 nm (35–37). Consistent with the relative polyP content observed with toluidine blue O staining (Fig. 1A), DAPI-stained polyP granules were not obvious in the strains with reduced polyP content (i.e., *vam6*Δ and *vtc4*Δ mutants) (Fig. 1B; Fig. S1B). Furthermore, a mutant lacking the endo- and exopolyphosphatases (Epp1 and Xpp1, respectively) accumulated a greater level of polyP and consistently had more granules (Fig. 1B, [34]). To determine the structural nature of the observed polyP granules, we stained wild-type cells with DAPI and the lipophilic dye FM4-64 (Fig. 1C). Our analysis revealed that polyP granules were localized within membrane-bound structures, suggesting that these granules are contained within vacuole-derived compartments, consistent with findings from previous studies (5, 30).

Considering the protective role of polyP against metal ion toxicity and oxidative stress (38, 39), we also tested the impact of Vam6 on growth on high levels of divalent metal ions (Fig. 1D). Consistent with our observations of differences in polyP content, we found that high levels of zinc, nickel, copper, calcium, and manganese negatively impacted the growth of the *vam6*Δ mutant, particularly at 37°C. The zinc sensitivity of the *vam6*Δ mutant was not observed at 30°C, compared to the marked sensitivity of the *vtc4*Δ mutant, and the latter mutant did not show marked sensitivity to other metals compared to *vam6*Δ mutants. The mutant lacking Epp1 and Xpp1 generally did not show sensitivity to any of the metals. The differences in sensitivity to various metals for the *vam6*Δ, *vtc4*Δ, and *xpp1*Δ *epp1*Δ mutants suggest that the *vam6*Δ mutant phenotypes were not directly related to impaired polyP accumulation. Additional contributions for Vam6, such as a role in the HOPS complex in support of vacuolar function, may account for the greater range of metal sensitivities. Taken together, these results reveal an influence of Vam6 on polyP homeostasis, possibly by participating in proper localization of the synthesis machinery and/or trafficking of this molecule.

### Another HOPS complex protein, Vps41, is also required for polyP accumulation

Endocytic trafficking and tethering complexes, such as HOPS and CORVET, regulate their distinct tethering activities through specific subunits unique to each complex. For the HOPS complex, these subunits include Vps39/Vam6 and Vps41, whereas the CORVET complex utilizes Vps3 and Vps8. Despite these differences, both complexes share a conserved set of core subunits—Vps33, Vps18, Vps16, and Vps11—which are critical to their structural integrity and function (10). Considering the polyP phenotype observed in the *vam6*Δ mutant, we further tested whether loss of the other HOPS or CORVET complex mutants had a similar phenotype. We found that cells deficient in the HOPS complex subunit Vps41 showed reduced levels of polyP by gel electrophoresis and did not contain obvious polyP granules, as seen with the *vam6*Δ mutant (Fig. S1). Interestingly, the loss of the Rab GTPase Ypt7, a Vam6/Vps39 interacting protein, also impacted the polyP levels in the cells (Fig. S2A). This result further underscores the functional connection between the HOPS complex and polyP regulation. In contrast, cells lacking the CORVET complex subunits Vps3 or Vps8 were less impaired and exhibited phenotypes more similar to wild-type cells (Fig. S1). Studies have demonstrated that human platelets contain dense granules, formed from mature multivesicular bodies, which serve as storage sites for polyP (25, 26). Given the critical role of the endosomal sorting complexes required for transport (ESCRT) in multivesicular body formation, we investigated whether the loss of an ESCRT complex would lead to defects in polyP homeostasis. However, our analysis of the *vps23*Δ deletion mutant revealed no significant reduction in polyP levels, suggesting that ESCRT complex disruption does not markedly affect polyP regulation under the conditions tested (Fig. S2B). In line with these observations, we tested polyP levels in the *vps39/vam6*Δ and *vps41*Δ deletion mutants of *S. cerevisiae* and found that both mutants exhibited a significant reduction of polyP (Fig. S2C and D). These observations further corroborate and indicate conserved connections between the HOPS complex and polyP. Overall, our findings highlight the pivotal role of the HOPS complex in polyP homeostasis, as evidenced by the significant reduction or absence of polyP in *C. neoformans* mutants lacking the Vam6/Vps39 or Vps41 subunits.

### Loss of Vam6 negatively impacts localization of a VTC complex subunit

**Fig 2:**
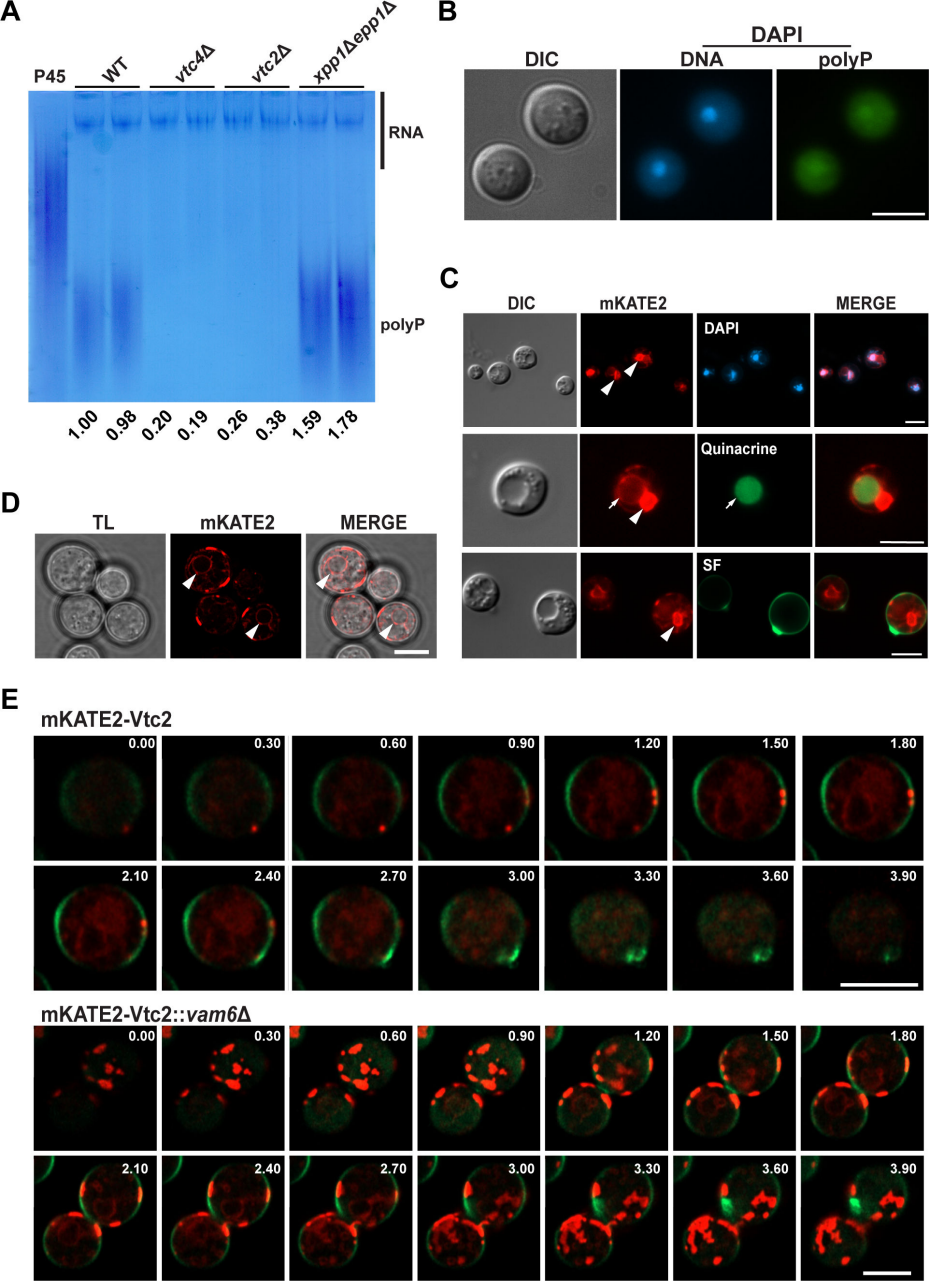
Vtc2 deletion reduces polyP content, and its localization is modulated by Vam6. (**A**) Detection of polyP on a native acrylamide gel stained with toluidine blue O. Total RNA extracts (10 µg) were from whole cell lysates of three biological replicates previously grown on YPD. The polyP types 45 and 700 (P45 and P700, 10 µg) were used as standards. The numbers indicate densitometry measurements of the regions containing polyP normalized to the wild-type control region. The acrylamide gel is representative of at least three independent experiments. (**B**) Wide-field fluorescence microscopy showing representative images of *vtc2*Δ deletion mutant cells from at least three independent experiments. Cells were stained and imaged as in Fig. 1B. Scale bar, 5 µm. (**C**) Wide-field fluorescence microscopy showing representative images of a wild-type strain expressing mKATE2-Vtc2 from two independent experiments, each observing more than 100 cells. Arrow and arrowheads indicating vacuole and perinuclear regions, respectively. Cells were stained as follows: DAPI (2 µg/mL–5 µg/mL) for nuclei detection, Quinacrine (200 µM) in HG3 buffer for vacuole detection, and the vital dye Solophenyl Flavine 7GFE (SF, 0.01%) for cell wall. Images were captured with (Ex/Em 359/46 nm) filter set for nuclei, (Ex, 572/25 nm; Em, 629/62 nm) filter set for mKATE2, and (Ex, 470/40 nm; Em, 525/50 nm) filter set for Quinacrine or SF. Differential interference contrast (DIC). (**D**) Micrographs show a *vam6*Δ deletion mutant expressing the mKATE2-Vtc2 construct (red), captured using laser scanning confocal microscopy. Arrowheads highlight perinuclear regions. Scale bar, 5 µm. (**E**) Serial stacks of merged laser scanning confocal micrographs of wild-type cells and *vam6*Δ deletion mutants expressing the construct mKATE2-Vtc2 (red) after staining with SF. Images were captured along the *z*-axis at 0.30 µm intervals, creating a series of optical sections for 3D reconstruction. Images were obtained with laser lines 561/488 nm and detected with Airyscan detector at: 422–497/607–735 nm for mKATE2, 420–480/495–550 nm for SF. Scale bars, 5 µm.

Recently, we found that polyP mobilization influences the formation of virulence factors and impacts the virulence of *C. neoformans* (28). Similarly, polyP also contributes to the virulence of bacterial pathogens, thus emphasizing the role of this molecule in infectious diseases (27, 40). Given the reduced level of polyP in the *vam6*Δ mutant and the absence of polyP granules, we hypothesized that the late endocytic trafficking mutants might have a role in the localization of the VTC complex. In *S. cerevisiae*, the biosynthetic and processing activities for polyP molecules involve VTC complex (subunits Vtc1, Vtc2 or Vtc3, Vtc4, and Vtc5) and exo- and endopolyphosphatases (41–43). The assembly and membrane localization of the VTC complex support the synthesis, vacuolar translocation, and storage of polyP in response to intracellular phosphate levels, the latter sensed via inositol signaling molecules and ATP (44–46). In *C. neoformans*, a recent study revealed that intracellular phosphate levels are also sensed through inositol pyrophosphate signaling molecules, particularly inositol pyrophosphate (IP7), which plays a pivotal role in regulating the phosphate signaling (PHO) pathway via its interaction with the SPX domain of Pho81 (46). Initially, we established that the Vtc2 subunit predicted for *C. neoformans* played a role in polyP synthesis by constructing and characterizing deletion mutants for the *VTC2* gene. Consistent with the *vtc4*Δ phenotypes that we previously characterized (34), the *vtc2*Δ deletion mutants exhibited reduced levels of polyP, absence of polyP granules, and sensitivity to high levels of zinc (Fig. 2A and B; Fig. S3). We next investigated if Vam6 influenced the localization of an mKATE2-Vtc2 fusion protein in the wild-type strain and in the *vtc2*Δ and *vam6*Δ mutants (Fig. 2B through E). We focused on Vtc2 because we were unable to obtain a functional fusion protein with Vtc4. Confirmation that the Vtc2 fusion protein remained functional was demonstrated by its ability to restore phenotypes of the *vtc2*Δ mutant to wild-type levels (Fig. S3). We observed that mKATE2-Vtc2 localized to cellular structures containing polyP (Fig. S3B), resembling the subcellular distribution of FM4-64-stained polyP granules (Fig. 1C), suggesting that Vtc2 is associated with the membrane of polyP-containing compartments. Wide-field fluorescence microscopy revealed that mKATE2-Vtc2 was detected in the perinuclear region, vacuoles, and at the cell surface in the wild-type strain (Fig. 2C and E). Our observations on mKATE2-Vtc2 distribution are consistent with previous studies on the intracellular localization of the VTC complex subunits in other organisms (47, 48). Interestingly, an aberrant distribution of mKATE2-Vtc2 was detected in the *vam6*Δ mutant (Fig. 2D and E). The protein primarily accumulated in patches along the cell periphery, yet it was also detected within the perinuclear region, resembling in part the distribution in the wild-type strain. These findings underscore the critical role of the VTC complex, particularly the Vtc2 subunit, in polyP synthesis and localization in *C. neoformans*. The aberrant distribution of mKATE2-Vtc2 in the *vam6*Δ mutant highlights the importance of late endocytic trafficking in maintaining the proper localization and functionality of the VTC complex, which is essential for polyP homeostasis and its broader impact on cellular processes.

### Loss of Vam6 impairs mitochondrial function and increases sensitivity to ETC inhibitors

**Fig 3:**
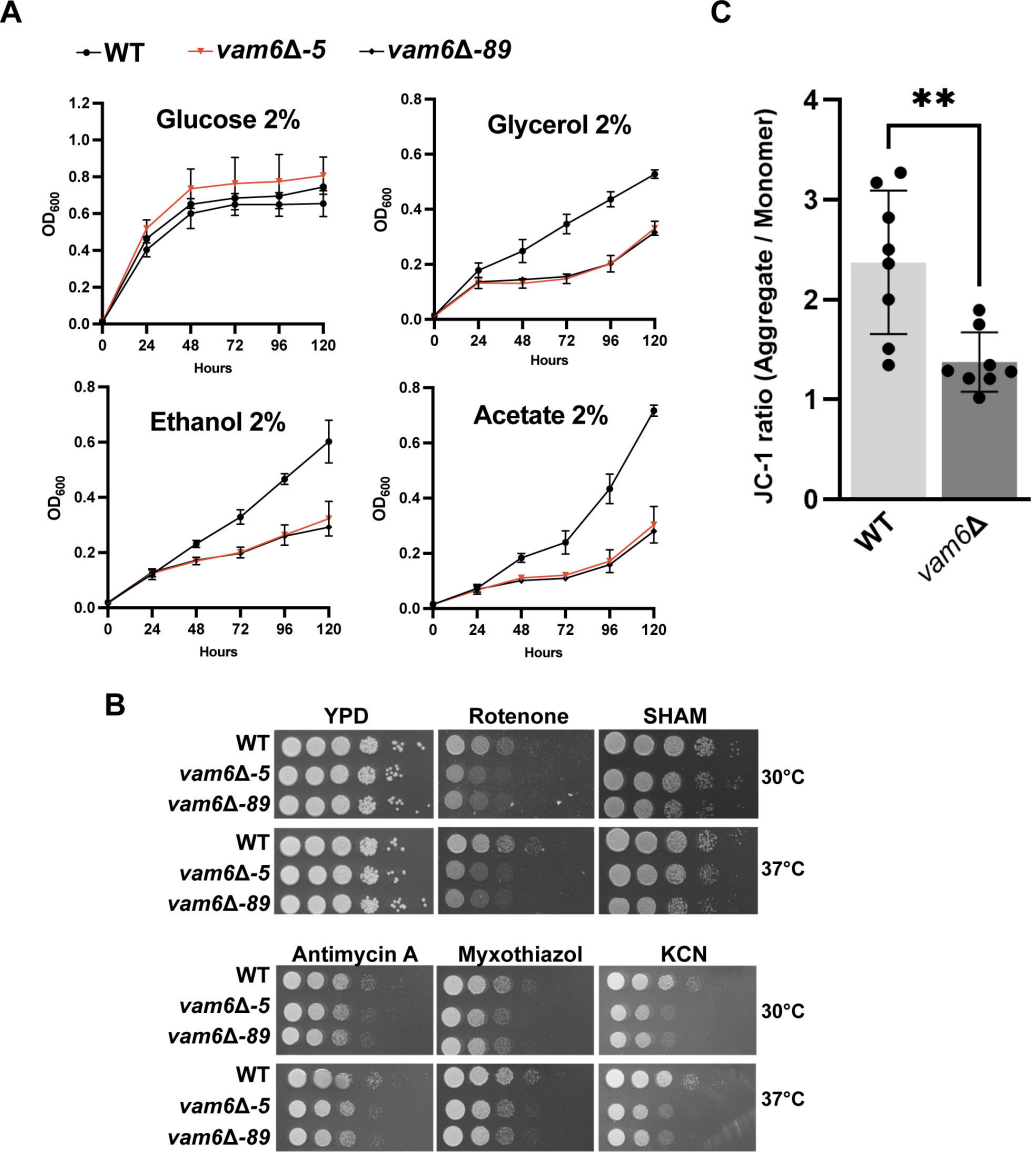
Trafficking mutants are impaired for mitochondrial function and exhibit increased sensitivity to ETC inhibitors. (**A**) Liquid growth assays on fermentative and non-fermentative carbon sources for the indicated strains. Cells were grown in yeast nitrogen base (YNB) without amino acids with 2% of glucose, glycerol, ethanol, or sodium acetate in 96-well plates. The plates were incubated at 30°C and 180 rpm for 120 h and optical densities (OD_600_) were taken every 24 h. The data represent the averages from three independent experiments ± standard deviation (SD). (**B**) Spot assays of 10-fold serial dilutions of the indicated strains onto solid YPD medium with or without rotenone (25 µg/mL), salicylhydroxamic acid (SHAM, 5 mM), antimycin A (5 µg/mL), myxothiazol (2.5 µM), or potassium cyanide (KCN, 10 mM). The plates were incubated at 30°C or 37°C for 2–3 days before being photographed. Images representative of at least three independent experiments. Note that the YPD plates without additions (30°C or 37°C) were also employed as controls for the experiment shown in Fig. 5C (labeled YPD + 0 mM H_2_O_2_). (**C**) Flow cytometry analysis for changes in the mitochondria membrane potential of the indicated strains. Measurements were performed on cells grown on YNB at 30°C for 16 h and stained with the mitochondrial dye JC-1 (5,5′,6,6′-tetrachloro-1,1′,3,3′-tetraethylbenzimi-dazolylcarbocyanine iodide, 2.5 µM) for 30 min at 30°C. The data represent the averages of the JC-1 ratios (aggregates/monomers) from three independent experiments ± SD. Statistical significance was assessed by unpaired *t*-tests (**, *P* < 0.01).

In addition to a role in the HOPS complex, Vam6 also is a key component of the vCLAMP membrane contact site between the vacuole and mitochondria. Considering the mitochondria-related functions of Vam6/Vps39 in yeast and mammalian cells, we hypothesized that loss of Vam6 might impair mitochondrial functions in *C. neoformans* (11, 49, 50). We therefore tested the growth of the mutants on non-fermentable carbon sources that require mitochondrial function to support respiration (Fig. 3A). We found that the *vam6*Δ deletion mutant exhibited a drastic growth reduction on media containing glycerol, ethanol, or acetate as carbon sources, suggesting that mitochondrial activity was impaired in the mutants. We also tested the growth of the mutants upon exposure to inhibitors of the mitochondrial ETC complexes (Fig. 3B). Consistent with the observed growth defects in respiratory conditions, we found clear sensitivity to inhibitors of mitochondrial complexes I and IV, and subtle sensitivity to inhibitors of complex III and alternative oxidase in the mutants. In general, defective vacuolar functions can negatively impact mitochondrial functions, leading to impaired energy production and increased cellular stress (11, 15, 50–54).

Mitochondrial membrane potential is also an important indicator of function, and changes in membrane potential occur in response to specific physiological conditions such as aging or stress (55–60). We therefore investigated the impact of the absence of Vam6 on the mitochondrial membrane potential using flow cytometry and the cyanine dye, JC-1. JC-1 fluorescence, upon accumulation in mitochondria, provides a quantitative ratiometric readout of polarized or depolarized organelles. Analysis of cells grown in minimal media showed that the membrane potential in the wild-type strain was twofold higher than in the *vam6*∆ mutant (Fig. 3C; Fig. S4). The reduced growth observed in respiratory conditions, together with the increased sensitivity to ETC inhibitors and the altered membrane potential, supports the conclusion that Vam6 influences mitochondrial function.

### Vam6 is required for enhanced polyP production upon ETC inhibition

**Fig 4:**
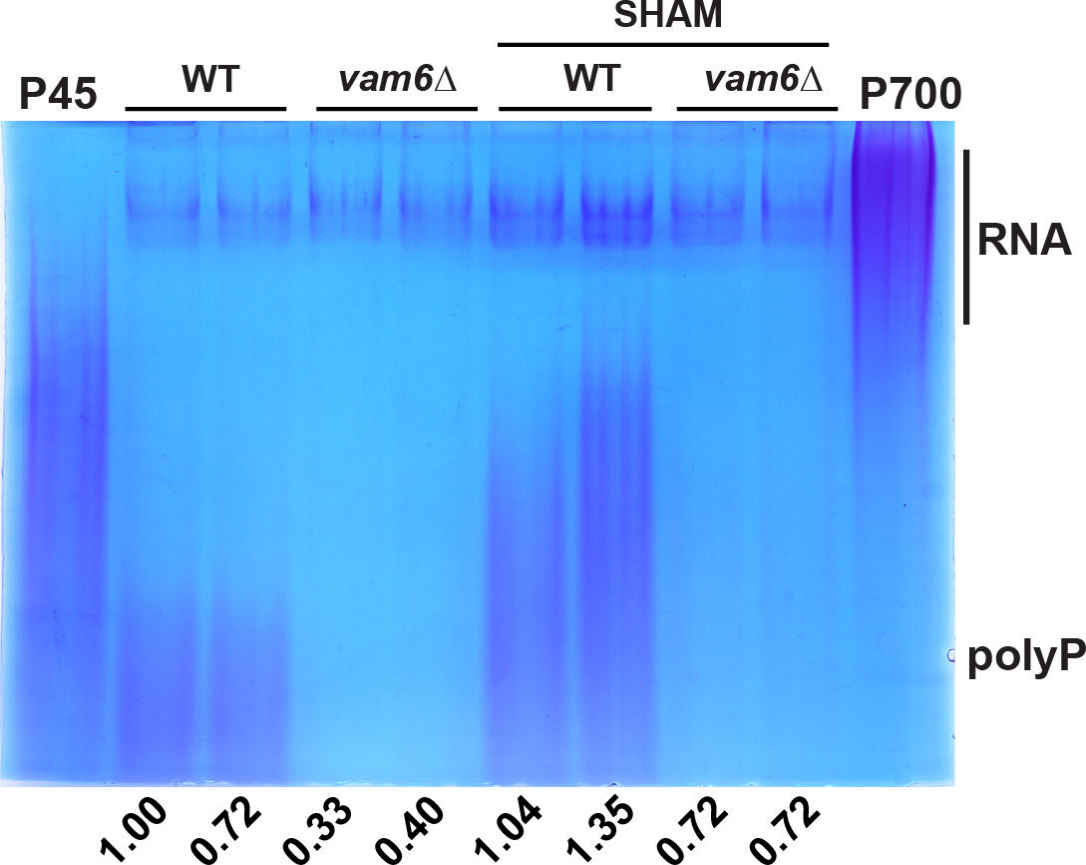
ETC inhibition has less influence on polyP homeostasis in a *vam6*Δ mutant. Detection of polyP of the indicated strains on a native acrylamide gel stained with toluidine blue O. Total RNA extracts (10 µg) from whole cell lysates of three biological replicates previously grown on YPD with or without SHAM (5 mM–10 mM). The polyP types 45 and 700 (P45 and P700, 10 µg) were used as standards. The numbers indicate densitometry measurements of the regions containing polyP normalized to the wild-type or untreated control regions. The acrylamide gel is representative of at least three independent experiments.

A role for polyP in the maintenance of mitochondria physiology has been identified in mammalian cells, where depletion negatively impacted ETC complexes and altered mitochondrial bioenergetics (61–63). A recent study in *C. neoformans* demonstrated that disruptions in the phosphate acquisition (PHO) pathway affect cellular levels of polyP and ATP, ultimately impacting energy metabolism, suggesting that polyP supplies phosphate for energy production (64). Similarly, in *S. cerevisiae*, polyP serves as a source of energy in the mitochondria and plays a crucial role in regulating energy metabolism (23, 65). In *C. neoformans*, disruption of phosphate acquisition and homeostasis leads to virulence defects and susceptibility to high levels of divalent metal ions (28, 34, 64). We hypothesized that defects related to mitochondrial function in the *vam6*∆ mutant (e.g., changes in membrane potential and the response to oxidative stress) could be related to changes in polyP metabolism or trafficking. We first tested whether ETC inhibition influenced polyP production and found enhanced levels, particularly upon treatment with the alternative oxidase (AOX) inhibitor salicylhydroxamic acid (SHAM) (Fig. 4; Fig. S5). This response is reminiscent of observations in *Phycomyces blakesleeanus* where levels of polyP were linked to the activity of the ETC (66). The influence is likely due to the protective function of AOX against oxidative stress (67–71). Interestingly, the influence of SHAM was partially blocked upon loss of Vam6, further linking the protein to mitochondrial function (Fig. 4).

### Vam6 influences sensitivity to oxidative stress

**Fig 5:**
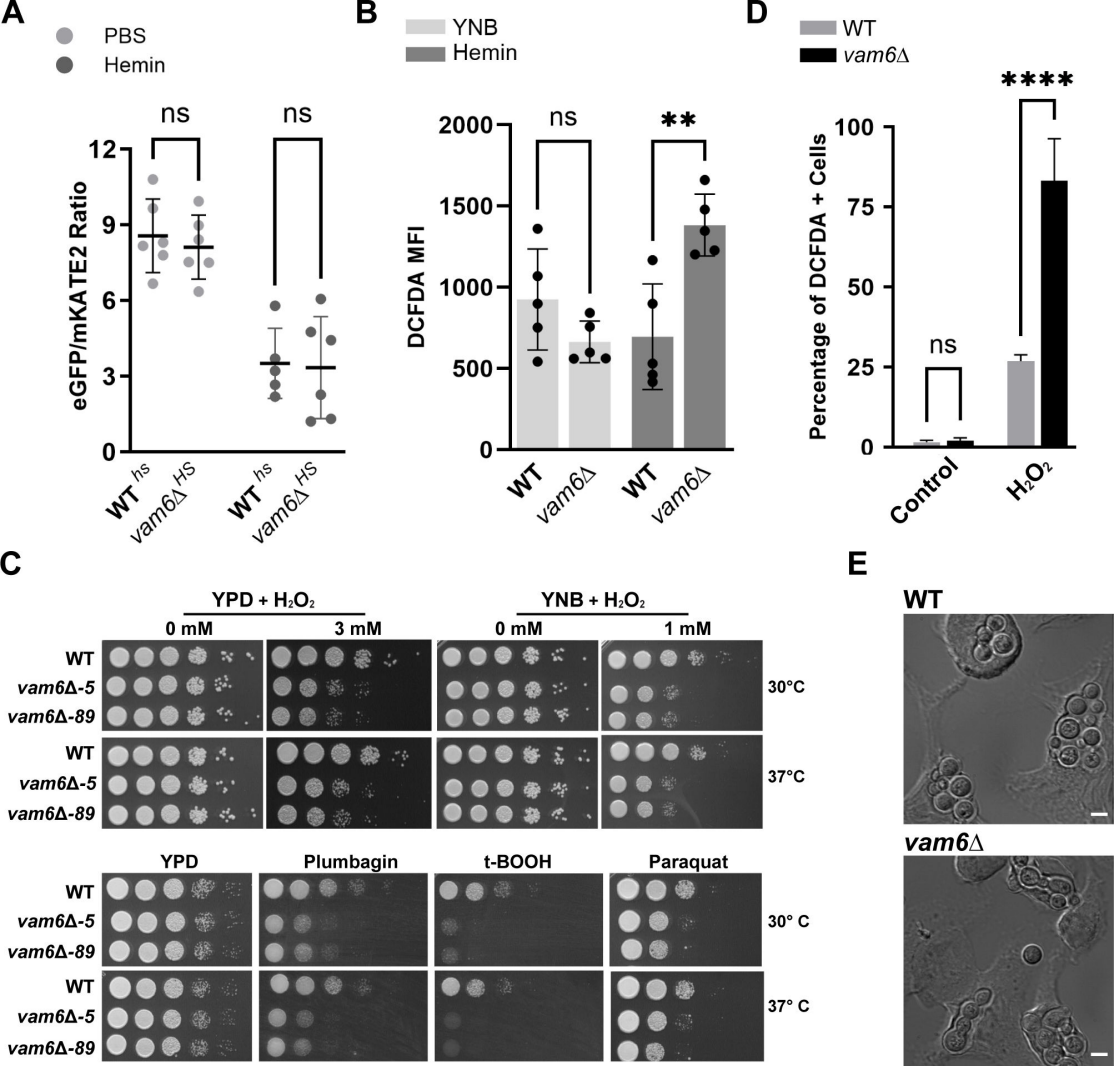
A genetically encoded heme sensor reveals no difference in cytosolic heme in the absence of Vam6, and Vam6 influences sensitivity to oxidative stress. (A) Changes of eGFP/mKATE2 ratios were measured by flow cytometry using fluorescein isothiocyanate (FITC) (525/40) and electron coupled dye (ECD) (600/20) filter sets, corresponding to the mean fluorescent intensities of eGFP and mKATE2 positive cells of the indicated strains expressing the heme sensor. The cells were iron-starved in dLIM supplemented with the iron‐chelator bathophenanthroline disulfonate (BPS) for 3 h and incubated in phosphate-buffered saline (PBS) with or without hemin (100 µM) for 1.5 h at 30°C. The results represent the averages from at least three independent experiments ± SD. Statistical analysis was performed using analysis of variance (ANOVA) followed by Bonferroni *post hoc* test (ns, not significant). (**B**) Flow cytometry analysis of intracellular ROS levels for the indicated strains. Measurements were performed on cells grown in yeast nitrogen base (YNB) with or without hemin (100 µM) for 16 h at 30°C using the cell-permeant ROS indicator dye 2´,7´-dichlorofluorescein diacetate (DCFDA, 16 µM) for 1 h. The results represent the averages from at least three independent experiments ± SD. Statistical significance shown was determined by ANOVA followed by Tukey *post hoc* test (**, *P* < 0.01; ns, not significant). (**C**) 10-fold serial dilutions of the indicated strains were spotted onto solid YPD or YNB supplemented with hydrogen peroxide (H_2_O_2_, 1 or 3 mM), plumbagin (100 µg/mL), tert-butyl hydroperoxide (t-BOOH, 350 µM), or paraquat (0.5 mM). The plates were incubated at 30°C or 37°C for 2–5 days before being photographed. Images representative of at least three independent experiments. Note that the YPD plates without additions (30°C or 37°C, 0 mM H_2_O_2_) were also employed as controls for the experiment shown in Fig. 3B. (**D**) Flow cytometry analysis showing cell percentages of DCFDA-stained populations. The indicated strains were grown to log phase in YPD and then treated with H_2_O_2_ (5 mM) for 1 h at 30°C. The results represent the averages from three independent experiments ± SD. Statistical significance was determined by ANOVA followed by Bonferroni *post hoc* test (****, *P* < 0.0001; ns, not significant). (**E**) Representative DIC micrographs of J774A.1 macrophage cells with internalized yeast cells of WT or *vam6*∆ strains at 24 h of interaction. Scale bars, 10 µm.

Previously, we demonstrated that deletion of the *VAM6* gene impaired the growth of *C. neoformans* on heme as the sole iron source (5). The mutant was also defective in the trafficking of iron uptake functions and the processing of Rim101, a transcription factor that influences the expression of iron uptake functions and growth on heme in *C. neoformans* (72, 73). Because of its oxidative and hydrophobic nature, heme can induce cytotoxic effects due to lipid peroxidation, DNA damage, and protein damage and aggregation if cellular homeostasis and trafficking are not properly regulated (74, 75). Given the potential roles of Vam6 in endocytic trafficking, we hypothesized that loss of the protein could alter heme acquisition or cellular heme homeostasis. To investigate intracellular heme levels in mutants lacking *VAM6*, we employed a codon-optimized, genetically encoded fluorescent heme biosensor (CnHS) for *C. neoformans* and performed ratiometric measurements of cytosolic levels of heme (5, 76). The heme biosensor encodes a bi-fluorescent molecule formed by the red fluorescent protein (mKATE2) n-terminally linked to green fluorescent protein (GFP), the latter containing a cytochrome *b-*562 heme binding peptide that quenches the GFP signal through Förster resonance energy transfer in a heme-dependent manner (76, 77). Strains expressing the heme sensor were grown to log phase and starved for iron in defined low iron media (dLIM) and then exposed to hemin for 1.5 h before performing flow cytometry measurements. Surprisingly, we found that the eGFP/mKATE2 ratiometric levels of the *vam6*Δ mutant were not significantly different from wild-type cells upon exposure to high hemin levels (Fig. 5A). This result suggests that Vam6 influences heme use by a mechanism that does not include trafficking to the cytosol.

To further examine whether defective homeostasis of heme induced oxidative stress in the *vam6*Δ mutant, we used flow cytometry to measure intracellular ROS upon treatment with hemin for 16 h and staining with 2´,7´-dichlorofluorescein diacetate (DCFDA), a cell permeant ROS indicator (Fig. 5B). We found that the *vam6*Δ mutant*,* upon treatment with hemin, exhibited a significant increase in intracellular ROS levels compared to the wild-type strain. This result suggests that trafficking defects caused by the absence of Vam6 likely enhanced intracellular ROS accumulation, potentially leading to accumulation of intracellular oxidative conditions.

Given the elevated ROS levels in the mutant, we further investigated the potential contribution of Vam6 in the oxidative stress response. We first performed serial dilution growth assays to test the sensitivity to ROS stressors and found that *vam6*Δ mutants showed a growth defect upon exposure to hydrogen peroxide (H_2_O_2_) in rich and minimal medium (Fig. 5C). We also found that the mutant strains were highly sensitive to ROS inducers such as plumbagin, hydroperoxide *tert*-butyl hydroperoxide (t-BOOH), and paraquat. Our results revealed that *vam6*Δ mutants showed sensitivity to all of the tested stressors, which contrasts with a previous study showing the absence of H_2_O_2_ sensitivity for a strain lacking Vam6/Vlp1p (33). We believe the difference may be mainly attributed to the stability of the reagents while performing the growth assays or differences in growth conditions. To further explore whether the observed sensitivity to H_2_O_2_ was indeed related to intracellular ROS levels, we performed flow cytometry using DCFDA (Fig. 5D; Fig. S6A). Consistent with the growth assays, high intracellular ROS levels were detected in the *vam6*Δ mutant after exposure to H_2_O_2_ in contrast to wild-type cells. We also confirmed that the DCFDA fluorescent signal was indeed caused by ROS accumulation and not by permeable dead cells (Fig. S6B and C). In a previous study, the vam6 deletion mutant (*vlp1*Δ) was shown to be essential for the proliferation of *C. neoformans* within macrophages (33). Based on our findings, it is plausible that ROS within the phagolysosome contribute to the reduced survival of the mutant cells. Consistent with this hypothesis, we observed morphological defects leading to chains of cells of the *vam6*Δ mutant inside J774 murine macrophages (Fig. 5E). Furthermore, similar to earlier reports, the survival of the mutant was compromised under these conditions (Fig. S6D). These results suggest that the loss of Vam6 renders *C. neoformans* highly sensitive to ROS, leading to impaired growth and survival under oxidative stress conditions.

The role of mitochondria in the adaptation to a variety of stress conditions has been reported in several fungal pathogens (16, 78–80). As introduced earlier, mitochondrial functions such as the ETC and mitochondrial morphology are associated with the response to host conditions during an infection with *C. neoformans* (16–18, 78, 81). Given the observed sensitivities to ROS stress of the mutants (Fig. 5), we first investigated whether alterations in mitochondrial morphology were present in the deletion mutants upon exposure to oxidative stress (H_2_O_2_). We utilized MitoTracker Orange and nonyl acridine orange (NAO) to study the impact of oxidative stress on mitochondrial morphology in the mutants. MitoTracker Orange allowed us to visualize active mitochondria based on membrane potential, while NAO provided insights into mitochondrial structures independent of their membrane potential. We found that the mutant strain showed no signal from MitoTracker Orange CMTMRos (MtrO) staining with or without H_2_O_2_ treatment, compared with detectable staining with NAO (Fig. S7A). This suggests the presence of depolarized mitochondria, regardless of the treatment. Furthermore, the mutant had reduced levels of tubular mitochondria and high levels of fragmented organelles compared with wild-type cells, which exhibited a mix of tubular, globular, and vesicular morphologies of active mitochondria (Fig. S7B). Taken together, these data reveal an influence of Vam6 on mitochondrial morphology/activity, with a potential impact on organelle function and the response to oxidative stress.

### Elevated mitochondrial ROS triggers increased polyP levels

**Fig 6:**
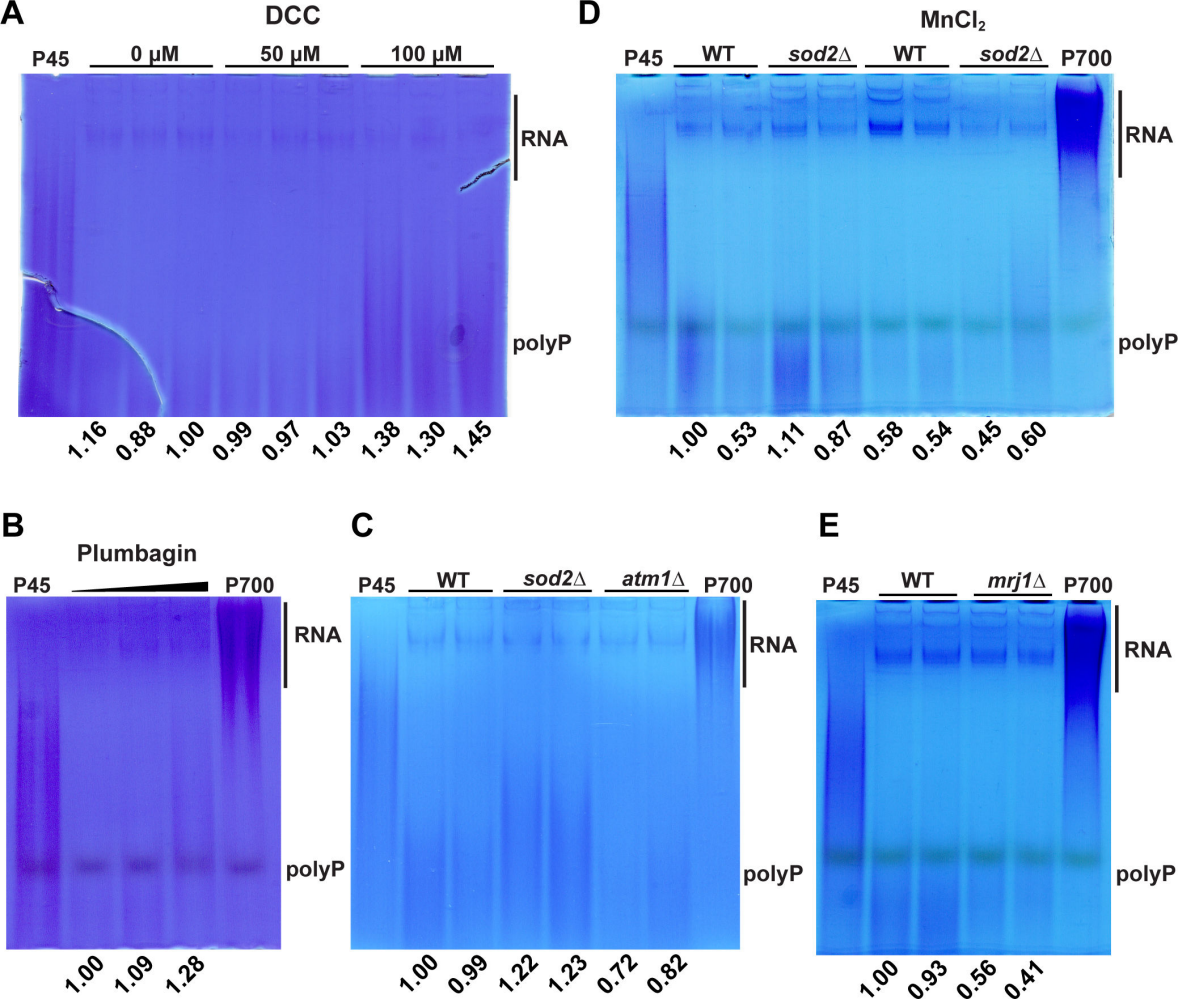
Dysregulation of mitochondrial ROS influences polyP homeostasis. (**A–E**) Detection of polyP on a native acrylamide gel stained with toluidine blue O for the indicated treatments of the WT strain and for the indicated mutants. Total RNA extracts (10 µg) from whole cell lysates of two to three biological replicates previously grown on YPD with or without DCC, plumbagin (0, 1, 2 µg/mL), or manganese chloride (MnCl_2_, 1.875 mM). The polyP types 45 and 700 (P45 and P700, 10 µg) were loaded as standards. The numbers indicate densitometry measurements of the regions containing polyP normalized to the wild-type control region. The acrylamide gels are representative of at least three independent experiments.

We next examined whether the influence of Vam6 on mitochondrial function and oxidative stress was connected with polyP. That is, we hypothesized that the reduced growth of the *vam6*Δ mutant upon mitochondrial inhibitors and its sensitivity to oxidative stress conditions might be related to the disruption of polyP homeostasis. PolyP plays key roles in mammalian physiological processes. These roles are linked to energy metabolism, calcium homeostasis, regulation of the mitochondrial transition pore, and the control of ROS metabolism (82, 83). Additionally, disruption of polyP phosphatase activity in the mitochondria has an impact on cell viability under oxidative stress. We found that inhibiting the mitochondrial ATP synthase with N,N´-dicyclohexylcarbodiimide (DCC) or incubation with plumbagin to provoke oxidative stress leads to increased polyP (Fig. 6A and B). Remarkably, we also observed a marked increase in polyP levels in the strain lacking the antioxidant mitochondrial superoxide dismutase 2 (*sod2*Δ) (Fig. 6C and D). The elevated polyP levels in the cells could be linked to oxidative conditions in the mitochondria, similar to the effects observed upon inhibition of AOX with SHAM (Fig. 4). To investigate this hypothesis, we treated cells with manganese because of its known antioxidant properties in yeast (84). Notably, incubating the *sod2*Δ mutant strain with manganese chloride resulted in a significant reduction in polyP levels (Fig. 6D). Moreover, we investigated whether the opposite effect occurred in the presence of low mitochondrial ROS, as observed in *mrj1*Δ deletion mutants (78). Indeed, we found that the *mrj1*Δ strain displayed a subtle reduction in polyP content compared to the wild-type strain (Fig. 6E).

### Vam6 influences lipid homeostasis

**Fig 7:**
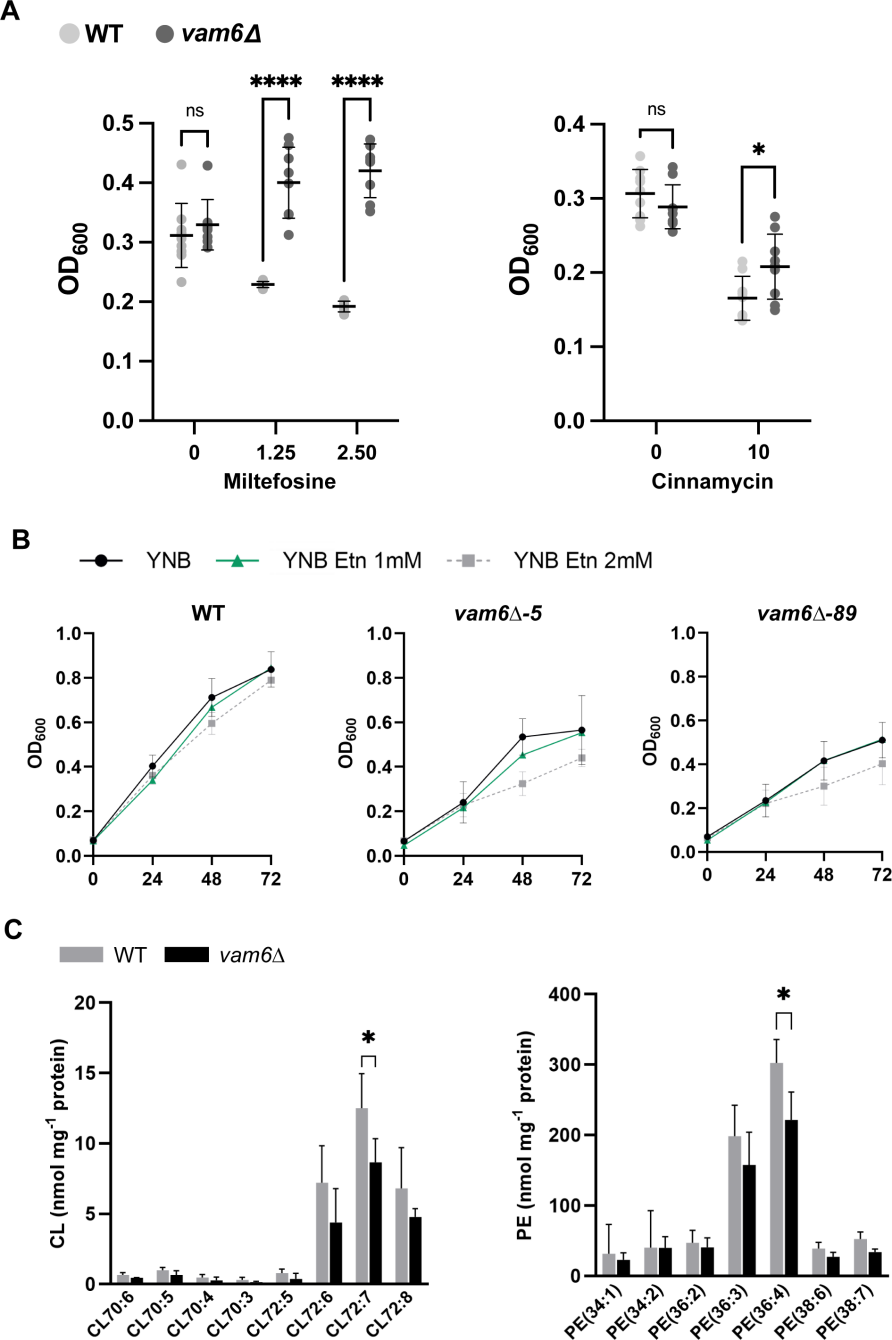
Trafficking mutants are impaired for phospholipid homeostasis. (**A**) Liquid growth assays of indicated strains grown in yeast nitrogen base (YNB) with or without phospholipid-binding inhibitors miltefosine (0, 1.25, and 2.5 µM) or cinnamycin (0 and 10 µM). Cells were grown in 96-well plates at 30°C and optical densities (OD_600_) were taken at 24 h of treatment. The data represent the averages from at least three independent experiments ± SD. Statistical significance was determined by analysis of variance (ANOVA) followed by Šídák’s multiple comparison *post hoc* tests (*, *P* < 0.05; ****, *P* < 0.0001; ns, not significant). (**B**) Liquid growth assays of the indicated strains incubated with YNB minimal media with or without ethanolamine (Etn, 1 and 2 mM). Cells were incubated in 96-well plates for 72 h at 30°C and 150 rpm–180 rpm. Optical densities (OD_600_) were taken every 24 h (*x*-axis represents time in hours). (**C**) Whole cell lipid analysis (LC-MRM/MS) showing the content of major species of PE and CL on the indicated strains. Cells were grown in YPD in the presence of mitochondrial inhibitor SHAM (3 mM) for 16 h at 30°C before being collected for lipid extraction. The data represent the averages from three independent experiments ± SD. Statistical significance was determined by ANOVA followed by Šídák’s multiple comparison *post hoc* tests (*, *P* < 0.05).

Defects in inter-organellar communication through MCS can interfere with the transfer of important molecules such as metal ions (e.g., calcium, iron) and the biosynthesis and transfer of phospholipids (85). Vam6/Vps39 is known to contribute to inter-organellar communication between mitochondria and the vacuole in different organisms (15, 86, 87). For example, a role for Vam6/Vps39 in the ethanolamine- or choline-stimulated trafficking of PE to the mitochondria was described in *S. cerevisiae* (12, 88). These findings prompted us to examine the role of Vam6 in phospholipid trafficking in *C. neoformans* in the context of the impaired growth of the *vam6*Δ mutant upon mitochondrial stress (5). Initially, we tested whether modifications in the phospholipid content impact growth of *vam6*Δ mutant upon exposure to cinnamycin and miltefosine, drugs that bind to and interfere with PE and phosphatidylcholine (PC), respectively (89–91). Miltefosine also has apoptotic properties in yeast impacting mitochondrial membrane potential (90, 91). Growth assays revealed that cinnamycin and miltefosine (more markedly) had reduced toxicity toward the mutant strain relative to the wild-type cells (Fig. 7A). Considering the reduced impact of phospholipid-binding drugs on the mutant, we also analyze the growth of cells upon supplementation of ethanolamine (Fig. 7B). We observed that minimal media supplemented with ethanolamine had a negative impact on the growth of the *vam6*Δ deletion mutants. These results prompted a further examination of the cellular PE and mitochondrial cardiolipin (CL) content of the *vam6*Δ mutant. This analysis was suggested by observations that phospholipids such as cardiolipins, PC, and PE influence mitochondrial membrane architecture to support the functions of ETC complexes (92–95). Moreover, regulation of phospholipid homeostasis is important for mitochondrial membrane adaptation to fluctuations of environmental conditions (96). We analyzed the lipid content by liquid chromatography-multiple reaction monitoring/mass spectrometry (LC-MRM/MS) for PE and by ultra-high performance liquid chromatography-multiple reaction montoring/mass spectrometry (UPLC-MRM/MS) for cardiolipin in wild-type and mutant cells after growth under mitochondrial stress conditions (Fig. 7C). We found that *vam6*Δ exhibited low levels of PE (36:4) and cardiolipin (72:7) in contrast to wild-type cells. Reduced levels of both phospholipid species in the mutant may contribute to impaired mitochondrial functions and cellular response to oxidative stress (12, 49, 94). More importantly, these phospholipid deficiencies might negatively impact the membrane integrity of the mutant, affecting ETC complex activity and assembly.

## DISCUSSION

In this study, we investigated the underlying mechanisms for the pleiotropic phenotypes of mutants lacking the Vam6/Vps39/TRAP1-domain protein Vam6. We discovered reduced polyP content in mutants lacking Vam6 and determined that a contributing mechanism may be a defect in functional localization of the VTC complex subunit Vtc2 for polyP synthesis. In *S. cerevisiae*, two sub-complexes, composed of Vtc1, Vtc4, and either Vtc2 or Vtc3, both share and have specific subcellular distributions and have been implicated in polyP production (48). Vtc proteins have additional roles in vacuolar membrane fusion, membrane trafficking, and microautophagy (97, 98). In *S. cerevisiae*, subunits of the complex have been detected in vacuolar membranes, in the perinuclear region, at the plasma membrane, and in cytosolic punctate structures (41, 47, 98). In *C. neoformans*, we hypothesize that the predominant perinuclear localization of Vtc2 reflects its association with the endoplasmic reticulum (ER), particularly the perinuclear ER. This localization may indicate a functional role in polyphosphate complex assembly, potentially serving as a precursor step before complex redistribution to other cellular compartments. Consistent with these observations, our finding that Vam6 and Vps41 mutants are impaired for polyP revealed that a functional tethering complex at late endocytic trafficking steps (e.g., at the vacuole) is essential for the correct subcellular distribution and activity of Vtc2 and presumably other polyP synthesis machinery. It is also possible that the complex is active at the cell surface or in internal trafficking compartments to generate extracellular polyP. Our previous demonstration of rapamycin sensitivity for *vam6*Δ mutants may also indicate a connection between Vam6, reduced accumulation of polyP, and the role of the VTC complex in microautophagy (98–100).

The observed variation in polyP content might account for the observed sensitivity of *vam6*Δ mutants to stress induced by high levels of divalent metal ions, as polyP is known to play a protective role in different organisms. For example, the sensitivity or tolerance to a wide variety of stressors has been linked to intracellular levels of polyP in bacteria (101). In fungal cells, polyP might also have a protective role in detoxification by forming complexes with divalent metal ions to prevent the generation of toxic hydroxyl radicals (27, 30, 34, 45, 102). We previously demonstrated a scavenging effect of polyP toward toxic metal ions such as zinc using mutants defective for the phosphate uptake system, the polyphosphate synthase Vtc4, and the exopolyphosphatase Xpp1 and Epp1 (27, 34). Our hypothesis of a potential role of Vam6 in polyP homeostasis is supported by our observations on the sensitivity of the *vam6*Δ mutant to zinc, nickel, copper, calcium, and manganese. Zinc toxicity has also been reported in a genome-wide screen performed in *S. cerevisiae* where over 100 genes were identified from defective mutants which accumulated and were distinctively sensitive to high levels of zinc (102). Interestingly, they found genes encoding proteins involved in phosphate homeostasis and proteins related to vacuolar protein sorting. The latter group of identified proteins, including the HOPS core subunit Vps33 and the SM protein Vps45, associated with autophagy and its possible role in the transport of excess zinc to the vacuole. We also note that Vam6 interacts with Ppn1 polyphosphatase in *S. cerevisiae,* thus indicating other potential connections between HOPS and phosphate (11). However, we note that the pattern of metal sensitivities for the *vam6*Δ mutants differed from that of the *vtc4*Δ mutant thought to be devoid of polyP. This observation suggests that loss of Vam6 has additional influences on metal ion sensitivity, likely through an impact on late endocytic processes and vacuolar function.

One independent function of the *S. cerevisiae* Vam6/Vps39 protein involves participation in vacuole-mitochondria membrane contact sites (MCS), so-called vCLAMPs (vacuolar and mitochondria patch), whose abundance is regulated by metabolic conditions (49, 86). Recently, we found that in *C. neoformans,* the Rab GTPase Ypt7, essential for vCLAMP formation in *S. cerevisiae*, may participate in vCLAMPs formation, and its cellular localization was linked to Vam6 or Vps41 (11, 103). This connection is interesting because mitochondria contain abundant levels of polyP in mammalian cells and are key players in oxidative stress (104). Consistent with this observation, we found that mutants lacking Vam6 exhibited a significant increase in intracellular ROS levels, perhaps in part due to altered polyP accumulation. Dysfunctional mitochondria have elevated ROS resulting in pleiotropic effects (16, 105). Here, we found that the absence of Vam6 caused growth defects in respiratory conditions, increased sensitivity to inhibitors of the ETC, and altered mitochondrial membrane potential and organelle morphology. Studies in *C. albicans* also demonstrate that Vam6 is required for oxidative stress tolerance and maintenance of mitochondrial and vacuolar functions, and it was suggested that Vam6 is necessary to keep proper pH balance under oxidative stress through an influence on vacuolar activity (15). It is also possible that sensitivity to ROS could be linked to disruption of autophagy caused by the absence of Vam6, and we previously demonstrated that the autophagy inducer rapamycin inhibited the growth of the *vam6*Δ mutant (5). The autophagy pathway is required to regulate redox homeostasis through different mechanisms, and the Vam6/Vps39 protein is involved in autophagy events. Interestingly, rapamycin inhibits the TOR pathway that regulates metabolism, respiration, and generation of ROS for mitochondria (106, 107). We note that a separate study analyzing the Vam6 deletion mutant (*vlp1*Δ) reported no growth defects under the tested conditions (33). However, in our assay, we identified sensitivity to multiple oxidative stress inducers, and the different results likely result from different assay conditions.

Considering the influence of Vam6 on mitochondrial activities and the response of the mutants to ETC inhibitors, we hypothesized that altered trafficking in the mutants disrupted the homeostasis of membrane phospholipids needed to support mitochondrial function. In *S. cerevisiae,* Vam6/Vps39 is involved in a HOPS or vCLAMP-independent mechanism related to PE trafficking between the ER and mitochondria when external ethanolamine is provided (12). It is also known that phospholipids such as cardiolipin and PE are important to support mitochondrial activities through the interaction with the various ETC complexes, and that the trafficking of phospholipids to and from mitochondria is crucial for the structure and function of the organelle (92, 94, 108–110). We suspected that the underlying causes of mitochondrial defects in the mutants might be related to disruption of phospholipid trafficking and/or their homeostasis. In fact, we found that under mitochondrial stress, the cellular levels of phospholipids were altered in the mutant. Moreover, reduced sensitivity to phospholipid-binding inhibitors was also apparent. Our observations are also consistent with the increased sensitivity to phospholipid inhibitors exhibited by deletion mutants (*cdc50*Δ) whose membranes have altered phospholipid levels (89). Recent findings on the regulation of vacuolar morphology through MCS and phospholipid homeostasis also highlight the importance of tethering complexes in intracellular organization and lipid metabolism (49, 111).

**Fig 8:**
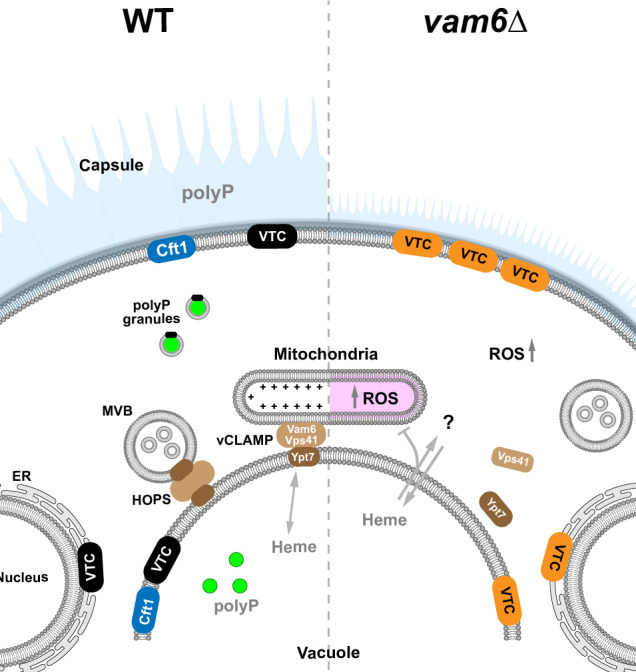
Summary model for the contributions of Vam6 to the localization/transport of the VTC complex and the interaction of vacuoles and mitochondria through membrane contact sites (vCLAMPs). *C. neoformans* Vam6 participates in the distribution of the VTC complex, including localization at the ER, cortical areas of the cell, and at the membrane of vacuoles, thus indirectly supporting polyP synthesis and/or the regulation of polyP homeostasis. The absence of Vam6 disrupts the recycling or assembly of the VTC complex at the cell surface, inducing the sequestration of the complex at the plasma membrane, potentially impacting the homeostasis/localization of polyP. The spatial dynamics of iron-related functions (Cft1) and heme transport/utilization at different organelles is also influenced by Vam6 (5). vCLAMPs support interchange of important molecules between the vacuole and mitochondria. The absence of Vam6 disrupts the fusion of vacuoles and multivesicular bodies (MVBs) and impairs mitochondrial activity by affecting the interaction between vCLAMP components Vps41 and Ypt7 (103, 112), which in turn interferes with the transport of essential molecules such as polyP, phospholipids, iron, and heme. In consequence, dysfunctional mitochondria lead to dysregulation of membrane potential (+) and ROS homeostasis.

In summary, we find that Vam6 has a dual role in regulating polyP metabolism in *C. neoformans* (Fig. 8). HOPS complex activity is required for proper localization of the VTC complex machinery, and vCLAMP activity modulates mitochondrial functions connected to polyP. An intriguing observation was that staining *C. neoformans* cells for polyP revealed localization in punctate bodies in the cytoplasm, and that co-localization was observed with mKATE2-Vtc2. The punctate bodies are reminiscent of the dense granules found to contain polyP in platelets (30). Defects in endomembrane trafficking impair dense granule formation, leading to a number of human disorders (25, 26). PolyP is a key procoagulant released by platelets to trigger fibrin formation and blood clotting (26). In this regard, we previously showed that loss of polyP in a *vtc4*Δ mutant of *C. neoformans* had a reduced ability to clot blood (34). These observations suggest that the analysis of polyP trafficking in *C. neoformans* may contribute more broadly to aspects of granule formation in mammalian cells. The absence of polyP in the *vam6Δ* mutant is unlikely to be the primary factor contributing to its virulence defect, as the pleiotropic effects of *VAM6* deletion likely play a significant role. Further studies are required to clarify the specific contributions of polyP to fungal virulence. A key analysis for future work will be to examine whether *C. neoformans* exports the polyP-containing punctate bodies as extracellular vesicles to exert an extracellular influence on the host.

## MATERIALS AND METHODS

### Strains and growth conditions

*C. neoformans* var. *grubii* H99 (serotype A) was used as the wild-type strain. Deletion mutants lacking Vam6, Vps41, Vps3, Vps8, Vtc4, or Xpp1 Epp1 and other proteins (Table S1) were generated from previous studies (5, 34, 113, 114). Maintenance of the strains, propagation, and serial dilution growth assays were performed in YPD rich agar medium (1% yeast extract, 2% Bacto‐Peptone, 2% D‐glucose, and 2% agar). For the growth assays in minimal media, plates were prepared with yeast nitrogen base (YNB, DIFCO) pH 5.6 or adjusted to pH 7.2 with 1M HEPES. For the analysis in low iron conditions, dLIM was prepared as described previously (76). Briefly, the following components were dissolved in low iron water (chelated with resin Chelex 100) and adjusted to pH 7.4: (5 g/L glucose, 5 g/L L‐asparagine, 0.4 g/L K_2_HPO_4_, 0.25 g/L CaCl_2_·2H_2_O, 0.08 g/L MgSO_4_·7H_2_O, 4.78 g/L HEPES, 1.85 g/L NaHCO_3_, 1 mL of 1,000× salt solution [0.005 g/L CuSO_4_·5H_2_O, 2 g/L ZnSO_4_·7H_2_O, 0.01 g/L MnCl_2_·4H_2_O, 0.46 g/L sodium molybdate, 0.057 g/L boric acid]). After filter sterilization, the media was supplemented with 0.4 mg/mL sterile thiamine and the iron‐chelator bathophenanthroline disulfonate (150 µM). All chemicals were from Sigma-Aldrich (St. Louis, MO) unless otherwise stated. For liquid growth assays to examine growth upon fermentative and non-fermentative carbon sources, or ethanolamine supplementation, cells previously grown in YPD were inoculated in YNB media without amino acids supplemented with 2% of glucose, glycerol, ethanol, or acetate and/or ethanolamine (1 or 2 mM) in 96-well plates. Phospholipid drug sensitivity tests in liquid media were performed in YPD or YNB with cinnamycin (10 µM; Cayman Chemicals 20136) or miltefosine (1.25 and 2.5 µM). All liquid growth assays were performed in 96-well plates at 30°C at 180 rpm for 48 to 120 h. Optical density (OD_600_) measurements were taken in a Tecan Infinite M200 Pro microplate reader.

### Flow cytometry analysis

Flow cytometric measurements were performed using the CytoFLEX S (Beckman Coulter) Flow Cytometer equipped with four laser lines (405, 488, 561, and 633 nm) fitted with filters for fluorescein isothiocyanate (FITC) (525/40), phycoerythrin (PE) (585/42), and electron couple dye (ECD) (600/20). The number of cells measured per experiment was set to 30,000–40,000. For the ROS sensitivity analysis, cells were grown with or without heme (100 µM) for 16 h at ~200 rpm and 30°C in YPD media and washed twice with PBS. Subsequently, cells were stained with DCFDA (16 µM) for 1 h at 30°C before measurements. For the ROS sensitivity analysis with H_2_O_2_, 2 OD cells were treated in YPD plus H_2_O_2_ (5 mM) for 1 h at 200 rpm and 30°C. After ROS treatment, cells were stained with DCFDA or propidium iodide (2.5 µg/mL) for 30 min at 30°C. For mitochondrial membrane potential measurements, cells were stained with the JC-1 dye (5,5′,6,6′-tetrachloro-1,1′,3,3′-tetraethylbenzimi-dazolylcarbocyanine iodide, Thermo Fisher Scientific, Waltham, MA, USA) (2.5 µM) for 30 min and 150 rpm. For the measurements of intracellular heme levels, cells expressing the cytoplasmic heme sensor were treated as described (76). Briefly, wild-type and deletion mutant strains expressing the heme sensor were iron-starved for 3 to 4 h at 30°C and ~200 rpm in dLIM and collected in tissue culture-grade phosphate-buffered saline (PBS). Subsequently, starved cells were treated with or without hemin (10 μM–100 μM) for 1.5 h at 30°C and kept on ice before measurements. eGFP and mKATE2 fluorescence were captured with FITC and ECD filter sets. Wild-type *C. neoformans* cells not expressing the heme sensor were used as a negative control for fluorescence. Analysis was performed with only mKATE2 positive cells. Data analysis and evaluation were conducted using FlowJo v.10.8 Software (BD Life Sciences). Statistical analysis was performed using GraphPad Prism software.

### Serial dilution growth assays

To examine *C. neoformans* wild-type or mutant strains, cells were grown in YPD or YNB for 16 h at 30°C. Precultures were washed and resuspended in sterile water, and 10-fold serial dilutions were performed from an initial concentration of 2 × 10^7^ cells per milliliter. Five microliters were spotted in solid YPD or YNB plates supplemented with different compounds and incubated at 30°C and/or 37°C for 2–5 days before being scanned. For the mitochondrial sensitivity response to inhibitors of the different electron transfer chain complexes, the following drugs were used: rotenone (25 µg/mL), SHAM (5 mM), antimycin A (5 µg/mL), myxothiazol (2.5 µM), or potassium cyanide (KCN, 10 mM). For the reactive oxygen stress response, rich (YPD) and minimal (YNB) media were supplemented with the following compounds: hydrogen peroxide (H_2_O_2_, 1 mM–3 mM), plumbagin (50 µM), tBOOH (350 µM), and paraquat (0.5 mM). To test the sensitivity to high levels of divalent metal ions, strains were grown in YPD with or without zinc chloride (ZnCl_2_, 2.5 mM), nickel sulfate (NiSO_4_ ·6H_2_O, 3 mM), copper chloride (CuCl_2_, 10 mM), calcium chloride (CaCl_2_, 0.25 M), or manganese chloride (MnCl_2_ ·4H_2_O, 1.875 mM).

### Determination of polyP content

Determination of the relative polyP content was performed on native acrylamide gels stained with toluidine blue O, as described (34). Briefly, cells previously grown overnight in YPD at 30°C were lysed in citrate buffer to release polyP and RNA. PolyP-RNA extracts (10 µg) were loaded onto native DNA polyacrylamide gels, using 10 µg of polyphosphate types 45 and 700 as standards (P45, Sigma-Aldrich, St. Louis, MO and P700, Kerafast, Boston, MA). After electrophoresis in 1× Tris-borate EDTA buffer, the gels were fixed with acetate and stained with toluidine blue O. Following acetate destaining, the gels were scanned. The densitometric analysis of toluidine blue O-stained polyP was performed using ImageJ software. Scanned gel images were imported into ImageJ, where the region beneath the RNA bands extending to the gel edge for each lane was selected and quantified to determine intensity values. To normalize the data, background intensity was measured from a blank area of the gel and subtracted from the band intensities. The normalized densitometric values were expressed relative to the control sample (wild-type or untreated), which was assigned a value of 1. Each experiment was conducted in triplicate to ensure reproducibility, and representative values are shown.

### Wide-field fluorescence microscopic detection of polyphosphate granules, mKATE2-Vtc2, and organelle detection

For visualization of polyP granules, cells were grown in YPD overnight at 30°C. Cells were washed in PBS three times and stained with DAPI (100 µg/mL) for 30 min at room temperature. After staining, the cells were collected and washed three times with PBS. Images were captured with DAPI (Ex/Em 359/461 nm) filter sets for DNA and the BrightLine (Ex/Em 407/530 nm) full multiband filter set for polyP. For organelle visualization, cells were stained as follows: DAPI (10 µg/mL–100 µg/mL) for 30 min at room temperature to detect nuclei, Solophenyl Flavine 7GFE (SF, 0.01%) for 10 min to detect the cell wall, and FM4-64 (Invitrogen, 5 µM) for membrane staining. For vacuole visualization, cells were washed once with HG3 buffer (HEPES/KOH 100 mM, glucose 3%; pH 7.5), stained with Quinacrine dihydrochloride (200 µM) for 10 min, then kept on ice for 5 min. Subsequently, cells were washed twice in HG3 and resuspended in the same buffer before imaging with a filter set (Ex, 470/40 nm; Em, 525/50 nm). mKATE2-Vtc2 imaging was performed using a filter set (Ex, 572/25 nm; Em, 629/62 nm). Fluorescence microscopy images were captured with the equipment described above using the ZEN Lite 2.3 (version 2.3.69.1000; Carl Zeiss) software and ImageJ 1.53q for image analysis.

### Confocal microscopy

For visualization of mKATE2-Vtc2, cells expressing the construct were grown and stained with DAPI and SF as previously mentioned. To obtain serial stack laser scanning confocal micrographs, we used a Zeiss LSM 980 confocal system coupled with an inverted Axio Observer Z1/7 microscope and a Plan-Apochromat 63×/1.40 oil DIC M27 objective lens. The Airyscan 2 detector was utilized in super-resolution mode, detecting at 422–497/607–735 nm for mKATE2 and 420–480/495–550 nm for SF with laser lines at 561 nm and 488 nm, respectively. For visualization of polyP granules and localization of mKATE2-Vtc2, a Zeiss LSM900 confocal system coupled with an inverted Axio Observer Z2 microscope and a Plan-Apochromat 100×/1.46 oil Ph3 M27 objective lens was used. The Airyscan detector was utilized in super-resolution mode, detecting at 575 nm–700 nm for mKATE2, 499 nm–592 nm for polyP, 400 nm–450 nm for DAPI, and 400 nm for bright field, respectively. Fluorescence microscopy images were analyzed using the ZEN Lite 2.3 (version 2.3.69.1000; Carl Zeiss) software.

### Lipid analysis

Cell lipid analysis was performed using LC-MRM/MS. Lipid extraction was performed by adding 100 µL of water and two 3 mm metal beads into cell pellets suspended in methanol (200 µL). Cell lysis was performed on an MM 400 mill mixer at 30 Hz for 2 min. Four hundred microliters of methanol and 300 µL of chloroform were then added. The samples were sonicated in a water bath for 2 min, followed by centrifugation at 21,000 *g* for 10 min. Clear supernatants were collected to a set of 1.5 mL Eppendorf tubes, and the protein pellets were used for protein assay using a standardized Bradford procedure according to the manufacturer’s user guide.

Serially diluted standards of lipid substances of PE (18:0/18:1(9Z)) were prepared in an internal standard solution of PE (17:0/18:1)-d5, dissolved in methanol-chloroform (1:1). After drying the sample supernatants under a nitrogen gas flow, the residues were dissolved in the same internal standard solution. Aliquots of each sample solution and each standard solution were injected into a C8 LC column (2.1 * 50 mm, 2.5 µm) to make two rounds of UPLC-MRM/MS on a Thermo Ultimate 3000 UHPLC system connected to a Sciex QTRAP 6500 Plus mass spectrometer operated in the positive ion mode for detection and quantitation of PE lipids. Chromatographic separations were performed with a mobile phase composed of ammonium acetate in water and acetonitrile-isopropanol (1:1). CL analysis was performed using ultra-high performance liquid chromatography-high resolution mass spectrometry (UPLC-HRMS). Briefly, the supernatant of each sample was dried, and the residue was dissolved in an internal standard solution of CL (14:0/14:0/14:0/14:0) in methanol-chloroform (1:1). Aliquots of the sample solutions and serially diluted standard substance solutions of CL (18:1/18:1/18:1/18:1) prepared in the same internal standard solution were injected into a C8 LC column (2.1 * 50 mm, 2.5 µm) to make UPLC-MRM/MS runs on a Thermo Ultimate 3000 UHPLC system connected to a Thermo Scientific LTQ-Orbitrap Velos Pro mass spectrometer operated in the negative ion Fourier transform mass spectrometry (FTMS) detection mode within a mass range of m/z 100 to 1,800 and at a 60,000 full width at half-maximum (FWHM) mass resolution. The chromatographic separation was performed with the use of a mobile phase of ammonium acetate solution and acetonitrile-isopropanol (1:2).

### Generation of the *C. neoformans* strains expressing the heme sensor and mKATE2-Vtc2

Two independent single *vam6*Δ deletion mutants were employed for the expression of a previously designed cytosolic heme sensor probe (CnHS) codon optimized for *C. neoformans* (76). Briefly, the vector (pESL018-2) containing the heme probe CnHS and the hygromycin selection marker for the targeted integration in *C. neoformans* was modified by replacing the selection marker with a neomycin marker (NEO). Subcloning of the NEO resistance gene was performed by PCR using the primers ES260 and ES261 from the pSDMA57 (safe haven) plasmid (115) and inserting the amplicon into the pESL018-2 vector previously amplified with primers ES262 and ES263. The generated vector (pESL018-3) containing the NEO resistance cassettes and the CnHS probe were sequenced and linearized with *Asc*I or *Pac*I for transformation of *C. neoformans* single mutant independent strains by the biolistic particle delivery method. Strains selected for their growth resistance to G418 disulfate salt were screened for genomic integrations of the constructs by PCR using the primers UQ2962, UQ3348, UQ1768, and UQ2963, as described elsewhere (115).

The mKATE2-Vtc2 construct was generated by introducing the Vtc2 gene into the pESL018-3 vector containing the mKATE2 gene. The *VTC2* gene and plasmid backbone without the heme sensor eGFP were amplified using primer pairs ES406-ES407 and ES402-ES403, respectively, and cloned in *Escherichia coli*. Subsequently, pESL018-3-Vtc2 was sequenced and utilized to transform via biolistic transformation into the indicated strains.

### Construction of gene deletion mutants

The single deletion mutants *vtc2*Δ were generated using the *C. neoformans* (KN99) knockout collection library obtained from the Fungal Genetics Stock Center (116, 117). Briefly, deletion cassettes were PCR amplified with primer pairs ES434-ES435 and ES408-ES409 from gDNA of Vtc2 deletion mutants, respectively. Purified cassettes containing the nourseothricin-resistance marker (nourseothricin acetyl transferase) were introduced using biolistic transformation, as described previously (118).

### Mitochondria morphology analysis

For the analysis of mitochondrial morphology, cells were grown in YPD overnight at 30°C. Precultures were washed in minimum media (YNB) twice and resuspended (2–4 OD) in YNB with or without H_2_O_2_ (1 mM–2 mM). Cells were incubated at 30°C for 1 h at 200 rpm. Mitochondrial morphologies were also evaluated in cells grown in tissue culture medium (Dulbecco’s Modified Eagle Medium (DMEM)) in the presence or absence of H_2_O_2_ for 0 and 24 h to induce oxidative stress. Subsequently, cells were collected and washed twice with PBS before staining with the mitochondria dyes MitoTracker Orange CMTMRos (Invitrogen, 200 nM) or Acridine Orange 10-nonyl bromide (Invitrogen, 200 nM) for 30 min at 30°C in the dark. After staining, the cells were collected and washed with PBS and kept on ice before microscopy observations. The morphological characteristics of organelles in treated cells were assessed following established criteria from previous studies (17, 18, 119). Mitochondrial morphologies were categorized as tubular (elongated tubules), globular, fragmented (small size), or diffused, as described earlier in cells under stress conditions. Differential interference contrast and fluorescence imaging were performed with a wide-field fluorescence microscope (Zeiss Axiovert 200) coupled with a CMOS camera (ORCA-Flash4.0 LT; Hamamatsu Photonics) along with a 100× oil immersion lens objective (numerical aperture, 1.40). The signals from MitoTracker Orange and Acridine Orange 10-nonyl bromide dyes were captured using filter sets (Ex, 572/25 nm; Em, 629/62 nm and Ex, 470/20 nm; Em, 500/25 nm, respectively), with exposure times ranging from 100 to 400 ms. For cell visualization and quantification, ZEN Lite 2.3 (version 2.3.69.1000; Carl Zeiss) software was used.

### Macrophage infection assays

J774A.1 murine macrophage-like cells (ATCC, Manassas, Virginia) were cultured in high glucose DMEM (Fisher Scientific) supplemented with 10% heat-inactivated fetal bovine serum and 2 mM L-glutamine (Gibco). The cells were utilized within 20 passages after thawing from liquid nitrogen. Macrophage cells were stimulated with 150 ng/mL phorbol myristate acetate (PMA) for 1 h before infection. Wild-type or mutant yeast strains were opsonized for 1 h using the monoclonal anti-glucuronoxylomannan antibody, clone 18B7. PMA-stimulated macrophages were infected with 2 × 10⁶ opsonized yeast cells at a multiplicity of infection of 10:1 (yeast:macrophage) for 2 and 24 h at 37°C with 5% CO₂. Non-internalized yeast cells were removed by washing with 1× PBS. Internalized yeast cells were quantified by lysing the macrophages with sterile distilled water, followed by plating the lysates on YPD agar to determine colony forming unit (CFU) counts at 2 and 24 h. For microscopy, J774A.1 macrophage cells were seeded onto eight-well chamber slides in DMEM and allowed to adhere overnight. Following adhesion, macrophage cells were washed and maintained in serum-free DMEM before being infected with opsonized yeast cells. At 24 h post-infection, cells were washed with 1× PBS, fixed with 4% formaldehyde in 1× PBS at room temperature for 10 min, and subsequently imaged.

## SUPPLEMENTAL MATERIAL

Fig. S1 mbio.00328-25-s0001.pdf Cells with defects in the CORVET and HOPS complexes show altered levels of polyP granules.

Fig. S2 mbio.00328-25-s0002.pdf *C. neoformans* strains lacking the vacuolar Rab GTPase Ypt7 and *S. cerevisiae* deletion mutants for the HOPS complex show altered levels of polyP.

Fig. S3 mbio.00328-25-s0003.pdf Expression of mKATE2-Vtc2 restores polyP levels and zinc sensitivity in Vtc2 deletion mutants.

Fig. S4 mbio.00328-25-s0004.pdf Gating strategy for the analysis of flow cytometry experiments.

Fig. S5 mbio.00328-25-s0005.pdf ETC inhibition influences polyP homeostasis.

Fig. S6 mbio.00328-25-s0006.pdf The loss of Vam6 increases ROS stress upon hydrogen peroxide exposure.

Fig. S7 mbio.00328-25-s0007.pdf Loss of Vam6 negatively impacts mitochondrial morphology independent of oxidative stress conditions.

Table S1 mbio.00328-25-s0008.pdf Primers, plasmids, and strains.

ASM does not own the copyrights to Supplemental Material that may be linked to, or accessed through, an article. The authors have granted ASM a non-exclusive, world-wide license to publish the Supplemental Material files. Please contact the corresponding author directly for reuse.
