# Supplementary material for: The HOPS and vCLAMP protein Vam6 connects polyphosphate with mitochondrial function and oxidative stress resistance in Cryptococcus neoformans
Source: mBio. 2025 Feb 25;16(4):e00328-25. doi: 10.1128/mbio.00328-25 (PMC11980578; doi:10.1128/mbio.00328-25)
Supplement: Fig. S1 — Cells with defects in the CORVET and HOPS complexes show altered levels of polyP granules. [file mbio.00328-25-s0001.pdf]

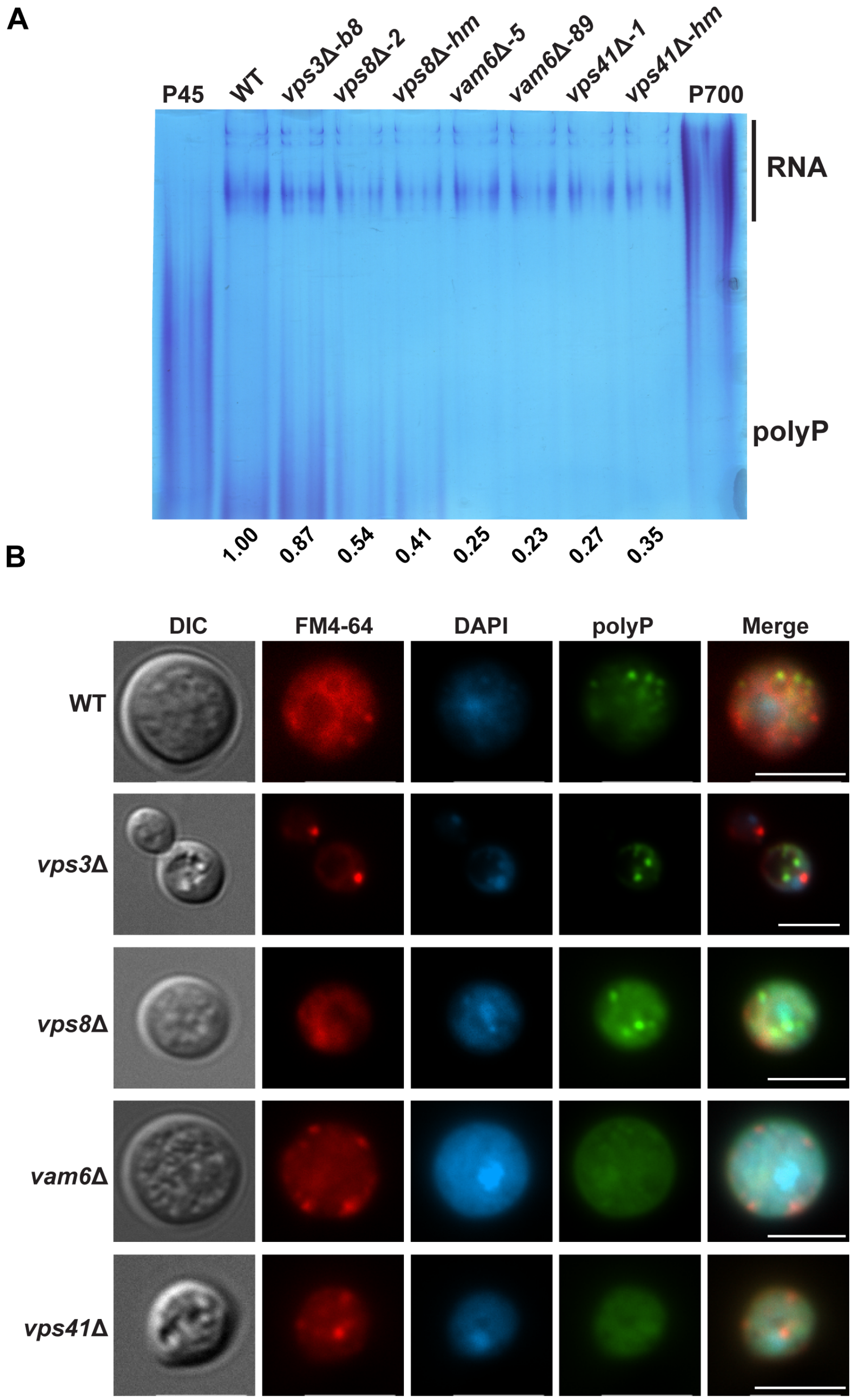

Supplemental Figure S1. Cells with defects in the CORVET and HOPS complexes show altered levels of polyP granules. A) Detection of polyP on a native acrylamide gel stained with toluidine blue O. Total RNA extracts (10 µg) from whole cell lysates of three biological replicates previously grown on YPD. Samples were loaded on the gels using polyP types 45 and 700 (P45 and P700, 10 µg) as standards. The numbers indicate densitometry measurements of the regions containing polyP normalized to the wild-type control region. The acrylamide gel is representative of at least three independent experiments. **B)** Wide-field fluorescence microscopy showing representative images of the indicated strains of three independent experiments, each observing more than 100 cells, stained with FM4-64 (5 µM) and DAPI (100 µg ml^{-1}) for 30 minutes at RT. Images were captured with DAPI filter set (Ex/Em 359/461nm) for DNA, BrightLine® full multiband filter set (Ex/Em 407/530nm) for polyP and Texas Red filter set (Ex, 572/26nm; Em, 645/45nm) for FM4-64. Differential Interference Contrast (DIC). Scale bars, 5 µm.
