# Supplementary material for: The HOPS and vCLAMP protein Vam6 connects polyphosphate with mitochondrial function and oxidative stress resistance in Cryptococcus neoformans
Source: mBio. 2025 Feb 25;16(4):e00328-25. doi: 10.1128/mbio.00328-25 (PMC11980578; doi:10.1128/mbio.00328-25)
Supplement: Fig. S2 — C. neoformans strains lacking the vacuolar Rab GTPase Ypt7 and S. cerevisiae deletion mutants for the HOPS complex show altered levels of polyP. [file mbio.00328-25-s0002.pdf]

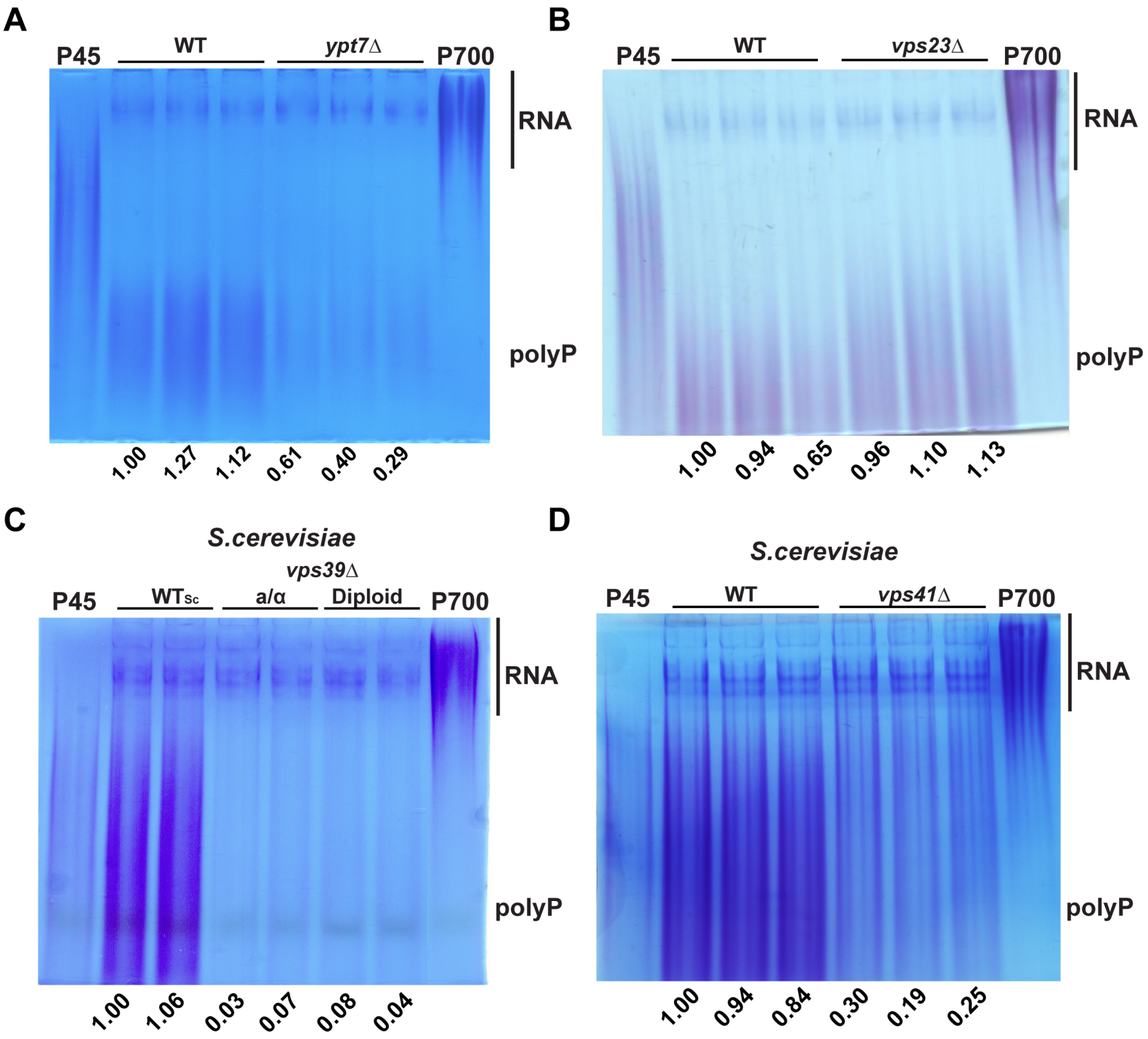

Supplemental Figure S2. *C. neoformans* strains lacking the vacuolar Rab GTPase Ypt7 and *S. cerevisiae* deletion mutants for the HOPS complex show altered levels of polyP. Detection of polyP of *C. neoformans* **(A, B)** and *S. cerevisiae* strains **(C, D)** on a native acrylamide gel stained with toluidine blue O. Total RNA extracts (10 µg) from whole cell lysates of three biological replicates previously grown on YPD. The polyP types 45 and 700 (P45 and P700, 10 µg) were loaded as standards. The numbers indicate densitometry measurements of the regions containing polyP normalized to the wild-type control region. The acrylamide gel is representative of at least three independent experiments.
