# Supplementary material for: The HOPS and vCLAMP protein Vam6 connects polyphosphate with mitochondrial function and oxidative stress resistance in Cryptococcus neoformans
Source: mBio. 2025 Feb 25;16(4):e00328-25. doi: 10.1128/mbio.00328-25 (PMC11980578; doi:10.1128/mbio.00328-25)
Supplement: Fig. S3 — Expression of mKATE2-Vtc2 restores polyP levels and zinc sensitivity in Vtc2 deletion mutants. [file mbio.00328-25-s0003.pdf]

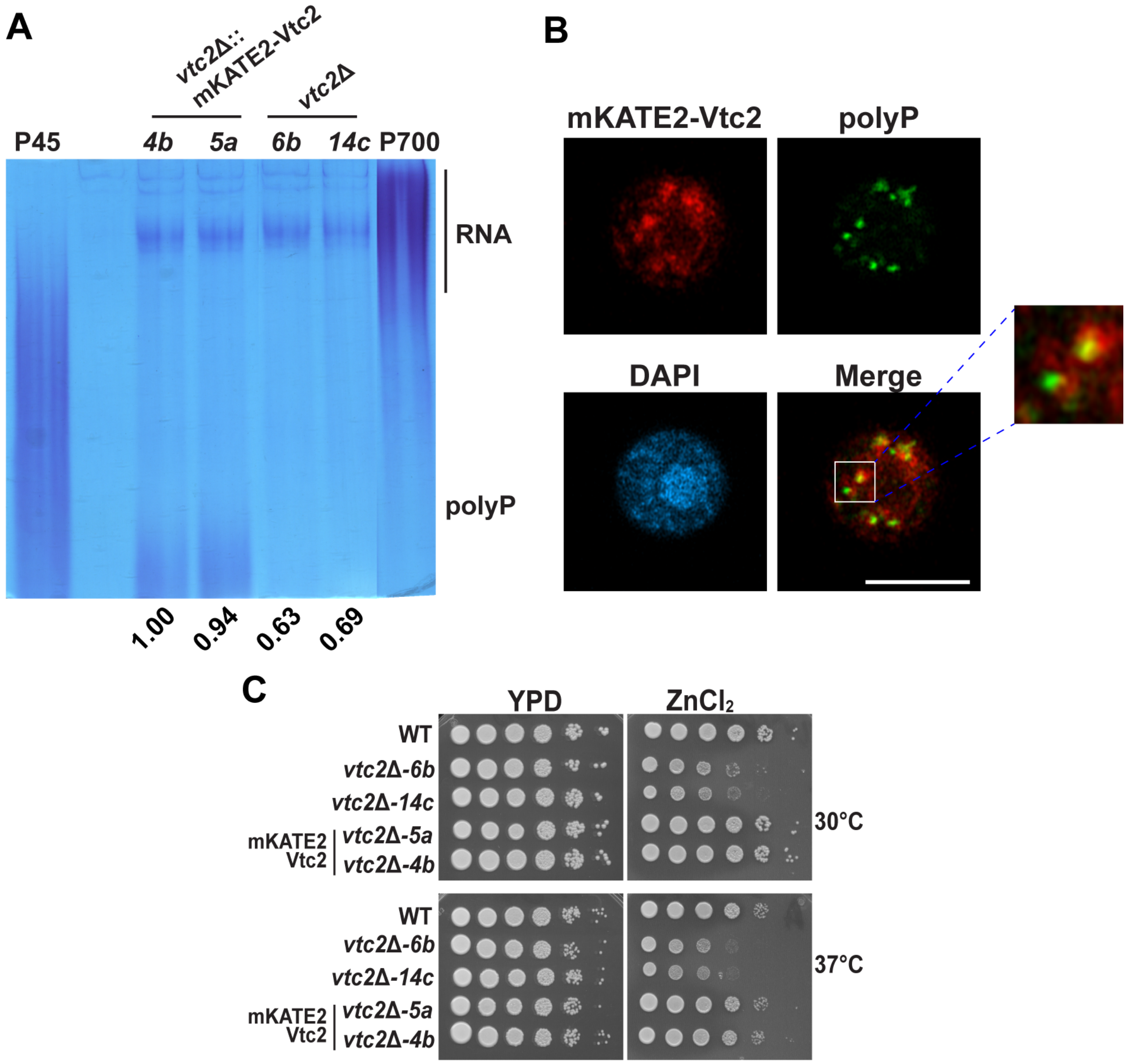

Supplemental Figure S3. Expression of mKATE2-Vtc2 restores polyP levels and zinc sensitivity in Vtc2 deletion mutants. A) Detection of polyP in independent *vtc2*Δ deletion mutant strains (6b, 14c) and cells expressing the *vtc2*Δ::mKATE2-Vtc2 construct (4b, 5a), analyzed using native acrylamide gels stained with toluidine blue O. Total RNA extracts (10 µg) from whole cell lysates of three biological replicates previously grown on YPD. The polyP types 45 and 700 (P45 and P700, 10 µg) were loaded as standards. The numbers indicate densitometry measurements of the regions containing polyP normalized to the complemented strain control (*vtc2*Δ::mKATE2-Vtc2-4b). The acrylamide gel is representative of at least three independent experiments. **B)** Laser scanning confocal microscopy showing representative images of a *vtc2*Δ deletion mutant strain expressing mKATE2-Vtc2 stained with DAPI (100 µg ml^{-1}) for 30 minutes and imaged as described in Figure 1B. Scale bar, 5 µm. **C)** Spot assays of 10-fold serial dilutions of the indicated strains onto solid yeast peptone dextrose (YPD) medium with or without zinc chloride ($ZnCl_2$, 2.5 mM). The plates were incubated at 30°C or 37°C for 2-3 days before being photographed. Images representative of at least three independent experiments.
