# Supplementary material for: The HOPS and vCLAMP protein Vam6 connects polyphosphate with mitochondrial function and oxidative stress resistance in Cryptococcus neoformans
Source: mBio. 2025 Feb 25;16(4):e00328-25. doi: 10.1128/mbio.00328-25 (PMC11980578; doi:10.1128/mbio.00328-25)
Supplement: Fig. S4 — Gating strategy for the analysis of flow cytometry experiments. [file mbio.00328-25-s0004.pdf]

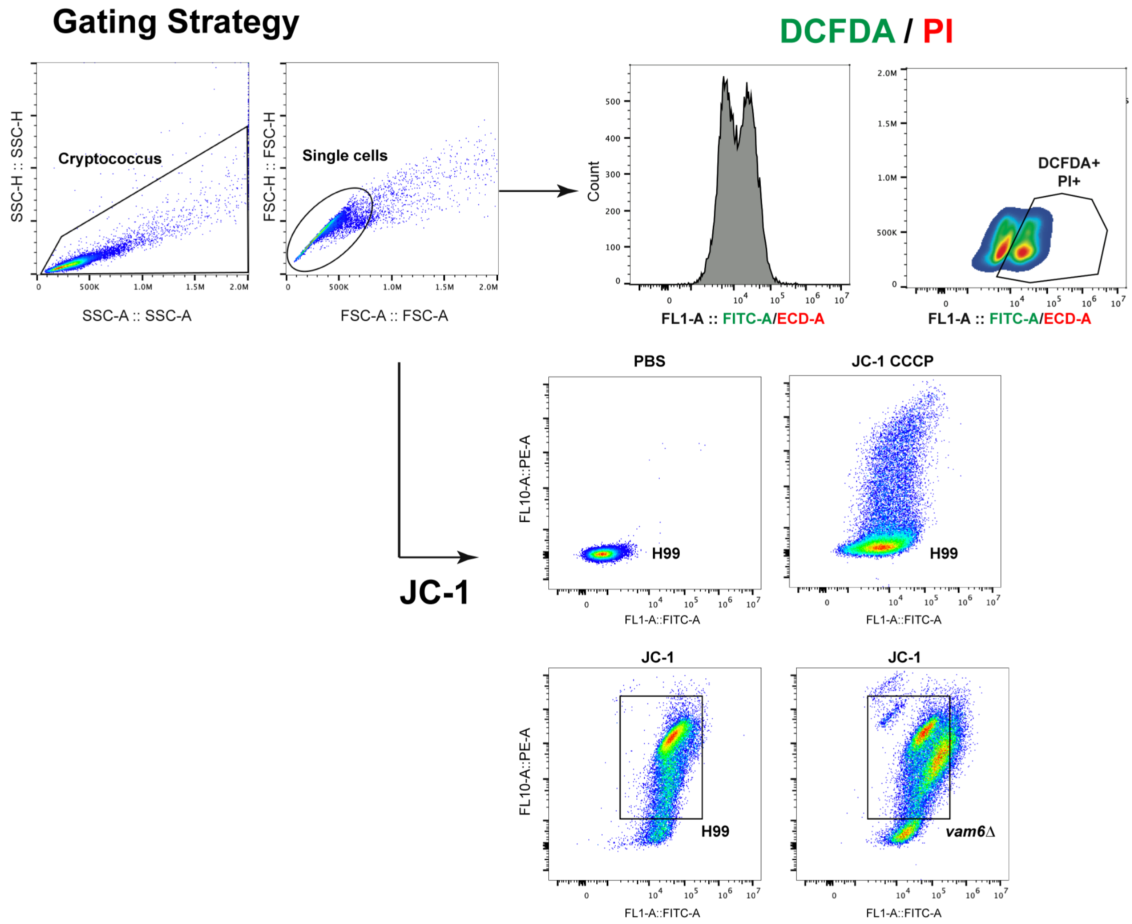

Supplemental Figure S4. Gating strategy for the analysis of flow cytometry experiments. Gating strategy for flow cytometry experiments performed in *C. neoformans* cells stained for intracellular ROS detection with DCFDA, for cell permeability with propidium iodide (PI), for mitochondrial membrane potential with JC-1 or with heme sensor ratio measurements (76).
