# Supplementary material for: The HOPS and vCLAMP protein Vam6 connects polyphosphate with mitochondrial function and oxidative stress resistance in Cryptococcus neoformans
Source: mBio. 2025 Feb 25;16(4):e00328-25. doi: 10.1128/mbio.00328-25 (PMC11980578; doi:10.1128/mbio.00328-25)
Supplement: Fig. S5 — ETC inhibition influences polyP homeostasis. [file mbio.00328-25-s0005.pdf]

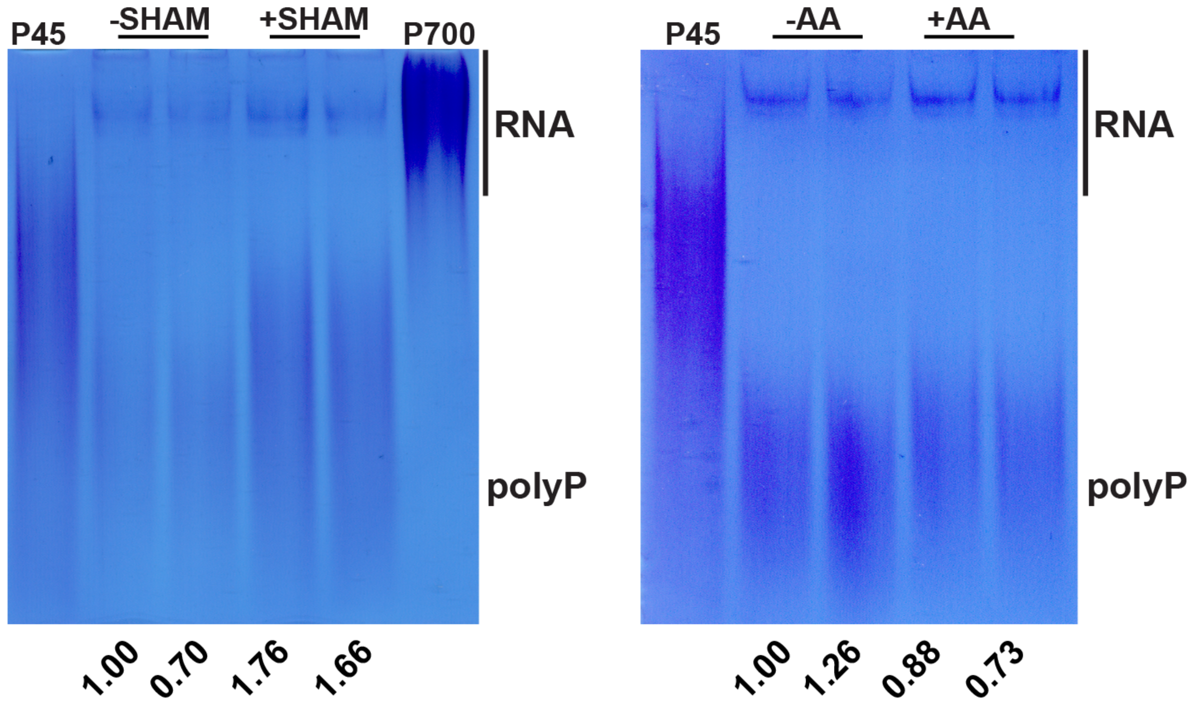

Supplemental Figure S5. ETC inhibition influences polyP homeostasis. Detection of polyP of wild-type cells (H99) upon treatment with ETC inhibitors on a native acrylamide gel stained with toluidine blue O. Total RNA extracts (10 µg) from whole cell lysates of two biological replicates previously grown on YPD with or without SHAM (5-10 mM) or antimycin A (5 µg/ml). Samples were loaded on the gels using polyP types 45 (P45, 10 µg) as standards. The numbers indicate densitometry measurements of the regions containing polyP normalized to the untreated control. The acrylamide gels are representative of at least three independent experiments.
