# Supplementary material for: The HOPS and vCLAMP protein Vam6 connects polyphosphate with mitochondrial function and oxidative stress resistance in Cryptococcus neoformans
Source: mBio. 2025 Feb 25;16(4):e00328-25. doi: 10.1128/mbio.00328-25 (PMC11980578; doi:10.1128/mbio.00328-25)
Supplement: Fig. S6 — The loss of Vam6 increases ROS stress upon hydrogen peroxide exposure. [file mbio.00328-25-s0006.pdf]

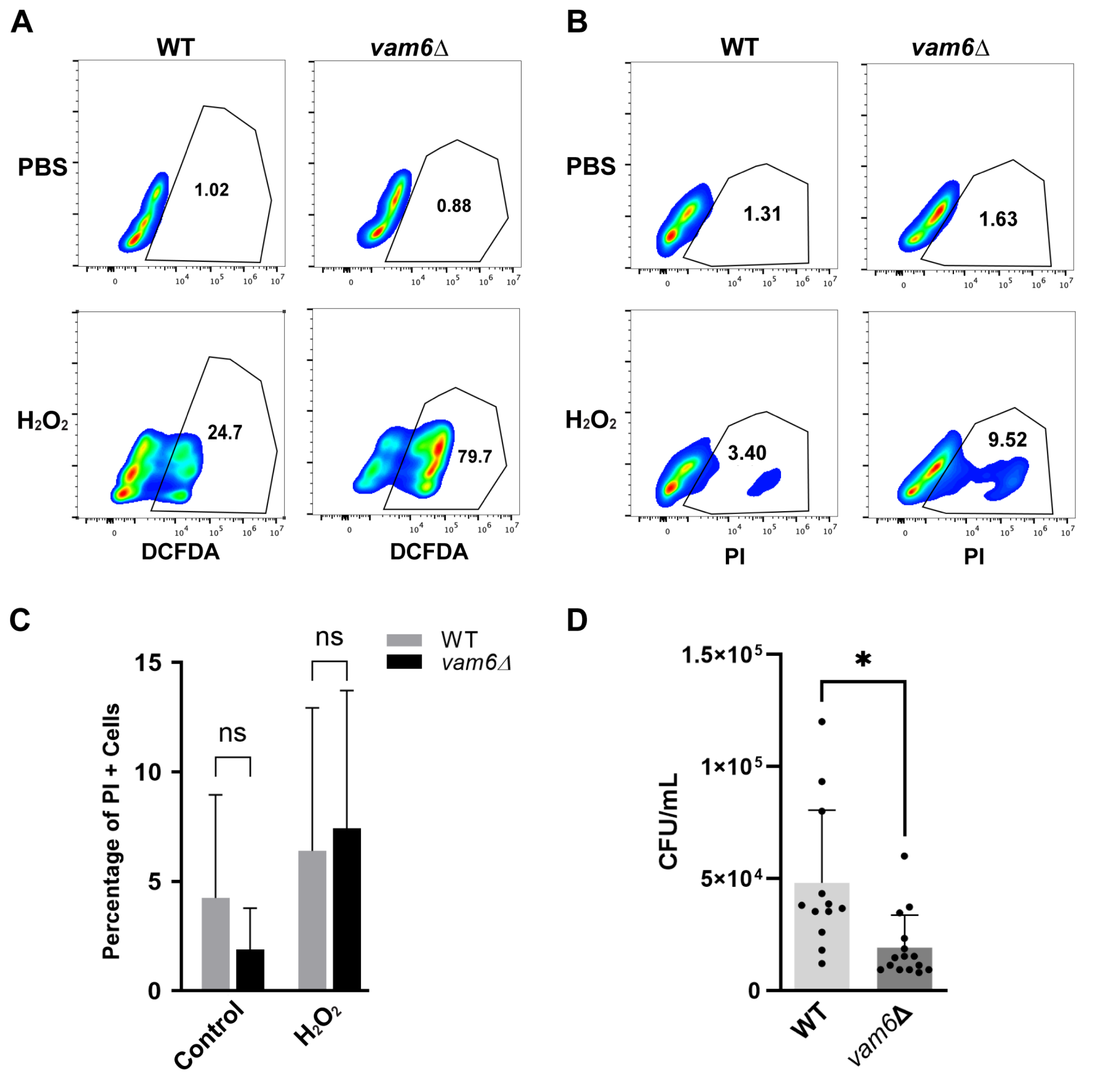

Supplemental Figure S6. The loss of Vam6 increases ROS stress upon hydrogen peroxide exposure. A) Dot plots showing DCFDA stained cell populations representative of the flow cytometry analysis (Figure 5D) of the indicated strains treated with or without H_2O_2 (5 mM) for 1 hour at 30°C. **B-C)** Flow cytometry analysis of the indicated strains showing dot plots and cell percentages of propidium iodide (PI) stained populations treated with or without hydrogen peroxide as in (A). The results represent the averages from three independent experiments ± SD. Statistical analysis was by ANOVA followed by Bonferroni *post hoc* test (ns: not significant). **D)** Survival of opsonized WT or vam6 strain in J774A.1 macrophages after 24 hours of interaction (Figure 5E). Statistical significance was determined by Kruskal-Wallis non-parametric test followed by Dunn's multiple comparison *post hoc* tests (*, P<0.05).
