# Supplementary material for: The HOPS and vCLAMP protein Vam6 connects polyphosphate with mitochondrial function and oxidative stress resistance in Cryptococcus neoformans
Source: mBio. 2025 Feb 25;16(4):e00328-25. doi: 10.1128/mbio.00328-25 (PMC11980578; doi:10.1128/mbio.00328-25)
Supplement: Fig. S7 — Loss of Vam6 negatively impacts mitochondrial morphology independent of oxidative stress conditions. [file mbio.00328-25-s0007.pdf]

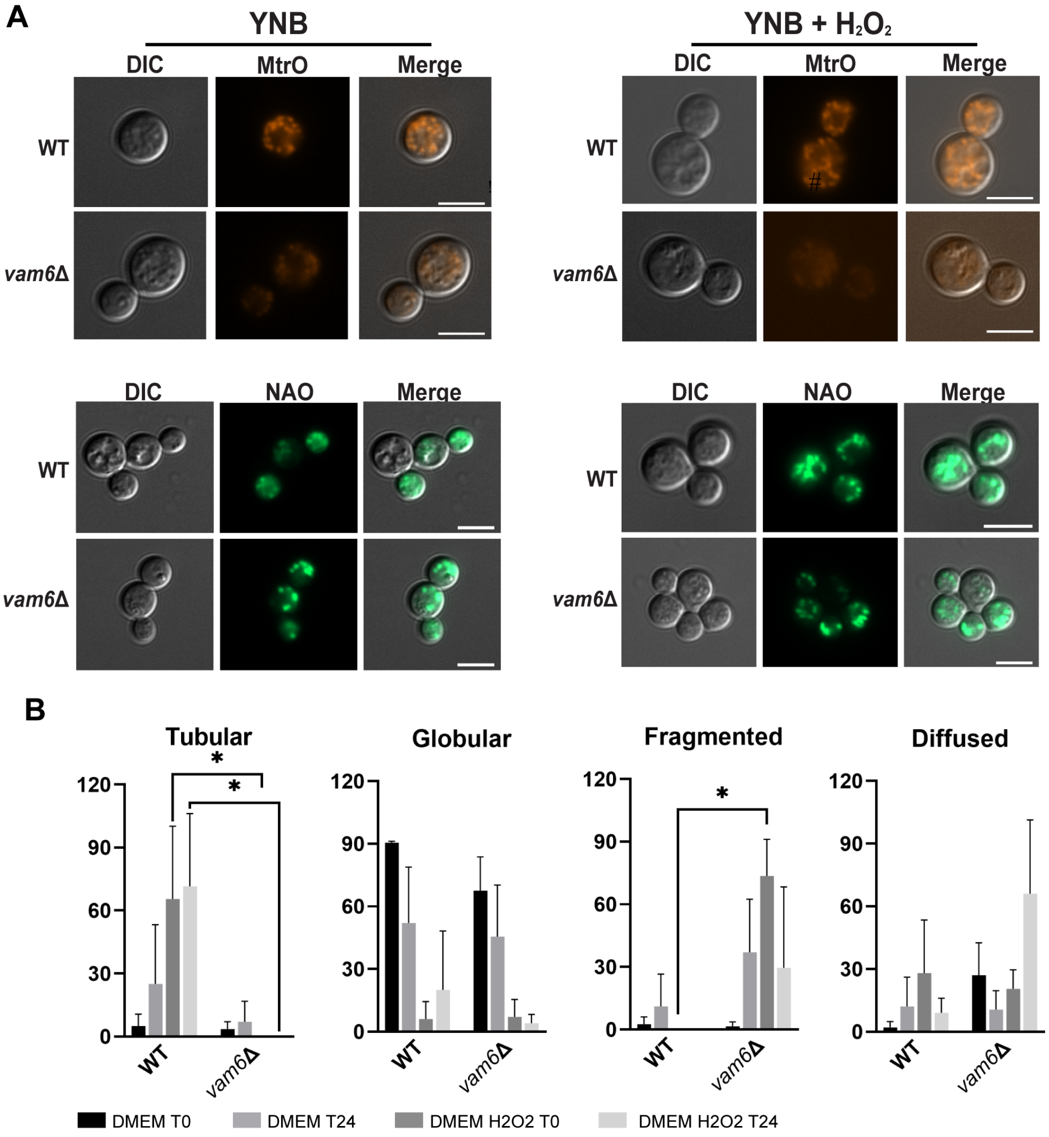

Supplemental Figure S7. Loss of Vam6 negatively impacts mitochondrial morphology independent of oxidative stress conditions. (A) Widefield fluorescence images showing mitochondrial morphologies of wild-type (H99) and *vam6*Δ mutant strains. The indicated strains were treated with or without H_2O_2 (1 mM) for 1 hour at 30℃ and then stained with the membrane potential dependent dye MitoTracker Orange CMTMRos (MtrO, 200 nM) or the mitochondria label nonyl acridine orange (NAO, 200 nM) for 30 min at 30℃ and 150-200 rpm. Images were capture with filter sets (Ex, 572/25 nm; Em, 629/62 nm) and (Ex, 470/20 nm; Em, 500/25 nm) for MtrO and NAO, respectively. The data are representative of three independent experiments, each observing more than 100 cells. Scale bars, 5 µm. **B)** Analysis of the indicated strains showing the percentage of cells with the different mitochondrial morphologies. Cells were grown and treated for 24 hours. Dulbecco's modified Eagle's medium (DMEM) with or without H_2O_2 (1 mM) at 37°C and 5% CO_2 before being stained and visualized as in (A). Data shown represent the average of three independent experiments representing at least 50 cells per time point for each treatment. Time (T) in hours. Statistical significance was determined by two-way ANOVA followed by Šídák's multiple comparison *post hoc* tests (*, P<0.05).
