# Supplementary material for: The HOPS and vCLAMP protein Vam6 connects polyphosphate with mitochondrial function and oxidative stress resistance in Cryptococcus neoformans
Source: mBio. 2025 Feb 25;16(4):e00328-25. doi: 10.1128/mbio.00328-25 (PMC11980578; doi:10.1128/mbio.00328-25)
Supplement: Table S1 — Primers, plasmids, and strains. [file mbio.00328-25-s0008.pdf]

Supplemental Table S1: List of primers, plasmids, and strains employed and developed during this study.

**Oligonucleotides**		
Primer Name	Sequence 5'→3'	Reference
ES260	ATGATTGAACAAGATGGATTGCACGC	This study
ES261	TCAGAAGAACTCGTCAAGAAGGCGATAGA	This study
ES262	TGACGAGTTCTTCTGAgtgaaggcggtaaggggtt	This study
ES263	CATCTTGTTCAATCATagacatgttgggcgagtttact	This study
ES402	agagccgcctccgccacccctgtgaccgagctta	This study
ES403	tgaCTCAGATCCCTAGATACATAAC	This study
ES406	tggcggaggcggctctATGAGTCAAGCAACAGACGC	This study
ES407	CTAGGGATCTGAGtcaGGAACTCTTGATGGCCGC	This study
ES408	ACCGTAATTCGGCCAAGGGTTC	This study
ES409	TCTTCGGAGCAAAAGAGCTTGC	This study
ES434	GACGGCGATTTTACGCACGCAT	This study
ES435	GAGTAAGCACCTGAATTCCTTG	This study
UQ2962	GGGTATGCCACAGATGCAGAT	(115)
UQ2963	TTGGATCCTCAATTGTCTCCT	(115)
UQ1768	TCAGCAACGCCGTTGAATCCT	(115)
UQ3348	ACTGGTGAGTACTCAACCAAG	(115)
Vam6-1	TGGCTGACTCTTGACCCACCTCGAGCT	(5)
Vam6-6	ACATGATCATGCCCATAGCCATAACATGCGA	(5)
**Plasmids**		
Name	Description	Reference
pESL018-2	Vector with the *C. neoformans* heme sensor (CnHS) for targeted integration	(76)
pSDMA25	Vector containing the safe haven region, a linker and nourseothricin resistance cassette.	(115)
pSDMA57	Vector containing the safe haven region, a linker and neomycin resistance cassette.	(115)
pESL018-3	Vector with the *C. neoformans* heme sensor (CnHS) for targeted integration harbouring the neomycin (NEO) resistance cassette.	This study
pESL018-3-Vtc2	Vector with the *C. neoformans* heme sensor (CnHS) containing *mKATE2-VTC2; pEF1-mKATE2-VTC2::NEO*	This study

pESL018-4	Vector with the *C. neoformans* heme sensor (CnHS) for targeted integration harbouring the NAT (nourseothricin acetyltransferase) resistance cassette	This study
**Strains**		
Name	Description, Genotype	Source, Reference
Wild type	*C. neoformans* H99 wild-type strain, serotype A	J. R. Perfect
vps3Δ-b8	*vps3* deletion mutant in the H99 background, *vps3Δ::HYG*	(5)
vps8Δ-2; vps8Δ-hm;	*Vps8* deletion mutant in: H99 background, *vps8Δ::HYG (-2) and* KN99 background, *vps8Δ::NAT(-hm)*	(5, 116)
vam6Δ-5, 89	*vam6* deletion mutant in: the H99 background, *vam6Δ::HYG.*	(5)
vps41Δ-2; vps41Δ-hm	*Vps41* deletion mutant in: H99 background, *vps41Δ::HYG (-1) and* KN99 background, *vps41Δ::NAT(-hm)*	(5, 116)
vtc4Δ	*vtc4* deletion mutant in the H99 background, *vtc4Δ::NEO*	(34)
xpp1Δepp1Δ-7	*xpp1* and *epp1* double deletion mutant in the H99 background, *xpp1Δ::NAT epp1Δ::NEO*	(34)
mrj1Δ	*mrj11* deletion mutant in the H99 background, *mrj1Δ::NEO*	(78)
sod2Δ	*sod2* deletion mutant in the H99 background, *sod2Δ::NEO*	(76)
WT^{hs}	*C. neoformans* wild-type strain expressing heme sensor, *pEF1-mKATE2-eGFP-*b_{562}*::HYG* targeted at safe haven.	(76)
vps23Δ	*vps23* deletion mutant in the H99 background, *vps23Δ::NEO*	(113)
ypt7Δ	*ypt7* deletion mutant in the H99 background, *ypt7Δ::HYG*	(103)
atm1Δ	*atm1* deletion mutant in the H99 background, *atm1Δ::NEO*	(114)
Wild type (sc)	*S. cerevisiae* SC288 wild-type strain	
vps39Δ-a/α	*vps39* deletion mutant in *S. cerevisiae* BY4743*, vps39Δ::KANMX*	Yeast Knockout Collection
vps39Δ-diploid	*vps39* deletion mutant in *S. cerevisiae* BY4741*, vps39Δ::KANMX*	Yeast Knockout Collection
vps41Δ	*vps41* deletion mutant in *S. cerevisiae* BY4741*, vps41Δ::KANMX*	Yeast Knockout Collection
mKATE2-Vtc2::H99	H99 wild-type strain expressing mKATE2-Vtc2 targeted at safe haven; *pEF1-mKATE2-VTC2::NEO*	This study
mKATE2-Vtc2::*vam6Δ*	*vam6* deletion mutant strain expressing mKATE2-Vtc2 targeted at safe haven; *pEF1-mKATE2-VTC2::NEO vam6Δ::HYG*	This study
mKATE2-Vtc2::*vtc2Δ*	*vam2* deletion mutant strain expressing mKATE2-Vtc2 targeted at safe haven; *pEF1-mKATE2-VTC2::NEO vtc2Δ::NAT*	This study
vam6Δ^{hs}	*vam6* deletion mutant in the H99 background with heme sensor, *vam6Δ::HYG, pEF1-mKATE2-eGFP-*b_{562}*::NEO*	This study
vtc2Δ -1a, -6b, 14c	*vtc2* deletion mutants in the H99 background, *vtc2Δ::NAT*	This study
